# Ecological contexts of diving behavior in Hawaiian false killer whales

**DOI:** 10.1186/s40462-026-00630-4

**Published:** 2026-03-06

**Authors:** Michaela A. Kratofil, Jacquelyn F. Shaff, Holly K. Hoffbauer, Mauricio Cantor, Marie C. Hill, Robin W. Baird

**Affiliations:** 1 Marine Mammal Institute, Department of Fisheries, Wildlife, and Conservation Sciences, Oregon State University, Newport, OR USA; 2 Cascadia Research Collective, Olympia, WA USA; 3 Marine Mammal Commission, Bethesda, MD USA; 4 University of Alaska Southeast, Juneau, AK USA; 5 Cooperative Institute for Marine and Atmospheric Research, Research Corporation of the University of Hawaiʻi, Honolulu, HI USA; 6 NOAA Pacific Islands Fisheries Science Center, Honolulu, HI USA

**Keywords:** Foraging, Diving behavior, Whales, Ecology

## Abstract

**Background:**

Predator movements vary across different ecological contexts, offering valuable insights into their foraging strategies. However, studying these contexts in marine predators is challenging due to the difficulty of observing them and their prey over sufficient spatiotemporal scales. Using bio-loggers and detailed life history information, we investigated abiotic and biotic factors shaping the diving behavior of a highly social apex predator—the false killer whale (*Pseudorca crassidens*)—around the Hawaiian Archipelago where three partially sympatric, genetically differentiated populations coexist.

**Methods:**

We deployed time-depth recorders (*n* = 5) and depth-transmitting satellite tags (*n* = 16) on false killer whales between 1999 and 2025 to describe diving at multiple spatiotemporal scales and ecological contexts. We fit generalized additive mixed-effects models to examine relationships between dive metrics and temporal and spatial proxies of prey distribution. Dive metrics were compared across demographic traits (sex, population, relative size) to assess potential drivers of behavioral variability.

**Results:**

False killer whales spent most of their time in near-surface waters and frequently dove within the epipelagic zone. Individuals exhibited various dive types within and among different habitats, including near-seafloor and deep ( > 1000 m; record maximum of 1424 m) diving behavior. Dive rates and depths were highest during daylight hours and full moons, although with significant inter-individual variation. Dive depth increased with current magnitude and mixed layer depth and decreased with lagged surface chlorophyll-a concentration. Larger individuals tended to dive deeper, although with high variation across demographic groups. These findings offer key insights into potential drivers of diving behavior, albeit with small effect sizes.

**Conclusions:**

We present the first comprehensive description of diving behavior for this species, which was characterized by variable temporal patterns, in contrast to sympatric species that are known to exploit diel vertically migrating prey. The diversity of dive types across habitats, along with trends between dive metrics and oceanographic variables, suggests that false killer whales may adjust their vertical movements to target different prey and environmental conditions.

***Hōʻuluʻulu Manaʻo (Hawaiian language abstract)*:**

***Ke Kahua*:**

ʻOkoʻa ka holo ʻana o ke poʻiiʻa ma nā ʻano honua kaiaola like ʻole, a hiki nō ke ʻike ʻia kā lākou kaʻakālai ʻai. Paʻakikī naʻe ke kilo ʻana i kēia mau honua o nā poʻiiʻa kai ʻoiai, paʻakikī ka nānā ʻana iā lākou me kā lākou ʻai ma nā pālākiō henua kaime. Ma o ka hoʻohana ʻana aku i nā lēpili ola a me nā ʻikepili ola kikoʻī, noiʻi mākou i hiki i nā mea ʻōnaeao a me nā mea ola kino ke pā i ke ʻano o ka luʻu ʻana o ke poʻiiʻa keu ma ka lauana ʻana - ke koholā ʻāhuka iwi poʻo like (*Pseudorca crassidens*) - a puni ka paeʻāina Hawaiʻi i noho pū ʻekolu pūʻuo ʻano pili a ʻokoʻa ma ke ōewe hoʻi.

***Kiʻina Hana*:**

Ua hoʻopaʻa mākou i nā mīkini ana kaime a hohonu (n=5) a me nā pepili hoʻoili ukali hohonu (n=16) ma nā koholā ʻāhuka iwi poʻo like mai nā makahiki 1999–2025 i mea e wehewehe aku ai i kaluʻu ʻana ma nā pālākiō henua kaime a me nā pōʻaiapii kaiaola. Ua hoʻohana mākou i nā ana pilina kaulike a me nā ana kaulele no ke kālailai ʻana aku i ka pilina wa ma waena o nā ʻikepili luʻu a me nā mea kaime a henua hoʻi o ka hoʻomalele ʻana o ka ʻai. Hoʻohālikelike ʻia nā ʻikepili luʻu ma nā waeʻanona ʻano (ke keka, ka pūʻuo, ka nui) i mea e kālailai ai i nā mea e hiki ke pā aku i nā ʻokoʻa o ka lawena.

***Nā Hua*:**

I ka hapanui o ko lākou ola, noho pinepine nā koholā ʻāhuka iwi poʻo like i ka ʻili kai a luʻu lākou i loko o ka wao mālamalama. Ma ka hoʻokahi, hōʻike ʻia nā ʻano luʻu like ʻole i loko o nā kaianoho ʻokoʻa, e like hoʻi me ka pili papakū a hohonu (>1,000m; ma ka hohonu loa 1,424m) lawena luʻu. ʻO ka pinepine a me ka hohonu o ka luʻu ʻana, ua kiʻekiʻe loa ma nā hola ao a me nā wā mahina poepoe, me ka loaʻa pū naʻe o ka ʻokoʻa o kēlā me kēia. Ua piʻi ka hohonu o ka luʻu ʻana i ka nui o ke au a me ka hohonu o ka wao pā lewa a ua iho i ka lohi o ka aea ʻana o ke kolopila-ʻā. ʻOi aʻe ka hohonu o ka piʻi ʻana o nā mea ʻoi aku o kona nui, eia naʻe, me ka nui o ka loli ʻana ma nā pūʻuo ʻano. Ma o kēia mau hua, loaʻa nō nā ʻike koʻikoʻi no nā e pā ana i ke ʻano o ka luʻu, i loko nō naʻe o ka liʻiliʻi o ka hopena.

***Nā Manaʻo Hope*:**

Hōʻike aku mākou ka wehewehena piha mua o ka hana luʻu o kēia ʻano lāhui, i kuhikuhi ʻia ma nā lauana kaime ʻokoʻa, i hoʻohālikelike ʻia me ka lāhui ʻano pili a ʻokoʻa ma ke ōewe hoʻi, kekahi lāhui i ʻike ʻia e luʻu a piʻi e ʻai i ka ʻai neʻena. Ma o ka ʻokoʻa o nā ʻano luʻu ma nā kaiaola, a me ke ʻano o nā ʻikepili luʻu a me nā ʻano mea kai, hoʻololi paha nā koholā ʻāhuka iwi poʻo like i ko lākou neʻena kū i mea e loaʻa ʻai kekahi ʻano ʻai ma nā ʻano pōʻaiapili kaiapuni like ʻole.

**Supplementary Information:**

The online version contains supplementary material available at 10.1186/s40462-026-00630-4.

## Background

Predators adopt foraging strategies that maximize resource gain and individual fitness [1]. Foraging decisions are informed by predator (e.g., morphology; [2]) and prey traits—type and size [3], mobility [4], and behavior [5, 6]. The physical environment interacts with these factors and shapes the availability, abundance, and patchiness of prey and competitors across different scales, thereby mediating the predator foraging strategy [7, 8] and movement decisions [9, 10]. For example, in resource-sparse or unpredictable environments, predators adopt flexible strategies such as covering large distances to locate dispersed prey patches (e.g., [11]) or adjusting movements to target diverse prey types (e.g., [12, 13]). Competition between species can also modulate foraging flexibility, with predators switching prey or habitat types to maintain success in the presence of competing specialists (e.g., [14]). The interplay between physical and ecological contexts therefore shapes individual predator decisions, which can scale up to population diversity and persistence, community dynamics, and ecosystem function [15–17]. However, understanding how these contexts modulate marine predator movements remains challenging due to the logistical difficulty of observing predators and prey over sufficient time and spatial scales [18].

In the marine environment, foraging occurs in three dimensions and thus predators must track the structuring of their prey through both horizontal and vertical movements (i.e., diving). Ocean physical properties are constantly in flux, forcing predators to effectively navigate the interactions between oceanographic and atmospheric conditions that shape biological productivity and patchiness across spatiotemporal scales [19–21]. Foraging in such environments is also limited by predator-specific physiological constraints (e.g., respiration in mammals and birds, [22]; thermoregulation in fish, e.g., [23]). These challenges result in diverse foraging strategies across marine taxa [19, 24]. For example, many marine predators closely track vertical migrations of prey that are known to vary over diel and lunar cycles (e.g., the deep scattering layer (DSL); e.g., rough-toothed dolphins, *Steno bredanensis,* [25], broadbill swordfish or aʻu ku, *Xiphias gladius,* [26]). Both static (e.g., seafloor topography, reefs) and dynamic habitat features (e.g., mesoscale eddies) can also promote variable foraging tactics among marine predators, and such tactics can be reflected in vertical movement patterns (e.g., [27–29]). Unraveling marine predator foraging strategies fills key behavioral and life history knowledge gaps in species that are hard to study—which is essential for interpreting their resilience to environmental fluctuations (e.g., [30])—and sheds light into the dynamics of ecologically and economically (i.e., for fisheries) important biomes [31, 32].

The false killer whale (*Pseudorca crassidens*) is a long-lived, highly social apex predator found in sub-tropical and tropical oceans worldwide [33, 34]. Most information on their ecology and behavior comes from long-term studies in the Hawaiian Islands where three genetically differentiated but partially sympatric populations are recognized: two resident to insular waters—Main Hawaiian Islands (MHI) and Northwestern Hawaiian Islands (NWHI) populations—and one open-ocean population ranging broadly offshore [35–39]. The MHI population is the most well-studied, within which four stable social clusters composed of relatives and regular associates have been identified [40–42]. Understanding false killer whale foraging strategies is crucial for conserving these populations, which face negative health effects from pollutant exposure [43, 44], harmful fisheries interactions (particularly for the MHI and open-ocean populations; [45, 46]), and reductions in prey size and quality [47, 48]. This is especially the case for the endangered MHI population (estimated at 139 individuals (95% credible interval: 114–162) in 2022), which has continued to decline at an annual rate of 3.51% since 2013 [49]. Identifying key foraging drivers can inform conservation strategies and mitigate cumulative threats to their survival. Based on visual observations and limited stomach content samples, false killer whales in Hawaiʻi appear to have a generalist diet, primarily feeding on large epipelagic game fish (e.g., tunas, dolphinfish or mahimahi, *Coryphaena hippurus*, wahoo or ono, *Acanthocybium solandri*) and some squid [33, 50]. Outside of these observations, the vertical movement behavior and foraging strategies employed by false killer whales in Hawaiʻi or anywhere are poorly understood (although see Minamikawa et al. [51] for 3-d of dive data).

Here, we advance the understanding of the vertical movement ecology of false killer whales around the Hawaiian Islands by assessing diving behavior collected with two different types of tags at different scales and across ecological contexts. Short-term Time-Depth-Recorders (TDRs) collect fine scale dive information at the expense of geolocation data and deployment longevity, while long-term satellite-linked depth-transmitting tags remain attached for longer durations, collect geolocation and summarized dive data, but at the expense of fine scale depth information. Together, data from these tags allowed us to address three objectives: (1) describe dive behavior to advance species-specific knowledge; (2) compare dive behavior across demographic groups and individual traits (e.g., population, sex, relative body size) to understand potential drivers of inter-individual variation; and (3) assess dive metrics in relation to temporal and environmental variables to generate hypotheses on strategies as they relate to potential prey behavior and distribution.

## Methods

All data processing and analysis detailed throughout were completed using the program R [52] unless noted otherwise. The ocean basemap image used in map figures is the intellectual property of Esri and is used throughout with permission (Copyright © 2025 Esri and its licensors, all rights reserved).

### Tagging procedures and programming

Tagging and small boat-based surveys throughout the main Hawaiian Islands took place from 1999 through 2025 as a part of a long-term study of odontocetes undertaken by Cascadia Research Collective (CRC; [53, 54]). When false killer whales were encountered, photographs were taken of all individuals present, and information on group size, behavior, and any foraging events were recorded. Tag deployments from NOAA Pacific Islands Fisheries Science Center (PIFSC) used the same methods, albeit tagging occurred during a large-scale ship-based false killer whale survey in 2024 [55].

Tags used to record diving behavior were attached in one of two ways. In the first part of the study (1999–2008), suction-cup attached time-depth recorder (TDR)/VHF radio tags (Mk6, Mk9, Wildlife Computers, Redmond, WA) were deployed on five individuals following methodology described in Baird et al. [56]. These tags sampled depth once per second at 1 m (Mk6) or 2 m (Mk9) increments (±1–2 m accuracy, respectively). We defined dives from the TDRs as periods when the individual went beyond two body lengths in depth (≥10 m). Maximum depth ranges of the tags were 250 m or 500 m, respectively, although due to temperature-related drift in the sensor this value is empirically shallower. Swim speed was also recorded by Mk6 tags (with a paddle wheel) and are presented as relative velocity (uncalibrated units) because readings vary with the position of the tag on the body [57] and likely with body size [56]. The TDRs used in this study do not record information on the geographic position of the animal, only dive depth (and relative velocity for Mk6 tags), although TDR-tagged individuals were tracked over some or all of the duration of tag attachments.

For the second part of the study (2010–2025), sixteen depth-transmitting satellite tags were deployed in the Low-Impact Minimally-Percutaneous External-electronics Transmitter (LIMPET) configuration [58] on or near the dorsal fin with two gas sterilized 6.7-cm surgical grade titanium darts, using a pneumatic projector. Tags were either SPLASH10 tags that transmitted dive behavior data and location data from Argos satellites or SPLASH10-F tags that transmitted the same data streams in addition to Fastloc®-GPS location data (hereafter, both referred to as “SPLASH” tags; Wildlife Computers, Redmond, WA). Programming regimes varied throughout the study period, but tags were generally programmed to optimize data transmission (e.g., based on satellite coverage); programming settings are provided in Additional File 1. Post-2013 deployments benefitted from one or more land-based Argos receivers (MOTEs; [59]) that enhance reception of behavior data.

Transmitted dive behavior from SPLASH tags was in the form of behavior logs, consisting of “dive” and “surface” periods summarized by the start and end times of each period, the duration of each period, the maximum depth reached during each dive, and the shape of the dive. Dive shape was categorized onboard the tags, defined by the total duration of the dive relative to the “bottom time” of the dive (i.e., any depth greater than or equal to 80% of the maximum dive depth; square = bottom time duration > 50%; u = bottom time duration > 20% but ≤ 50%; v = bottom time duration ≤ 20%). Dives were defined as any submergence greater than the user-programmed depth threshold (0–5 m; see Additional File 1), but the tags were programmed to ignore dives shallower than an additional depth and duration threshold to characterize deep dive behavior while optimizing battery life and minimizing gaps in the behavior data (i.e., increasing likelihood of reception of archived data by satellites). These latter two thresholds varied throughout the study period as lessons were learned from different programming regimes (Additional File 1). Any submergence shallower and shorter than these pre-defined depth and duration thresholds were considered “surface” periods. To maintain consistency in our analyses given the variable dive settings across tags, we considered dive records shallower than 50 meters and shorter than two minutes in duration as surface periods. Surface periods thus include shorter and shallower dives (e.g., inter-ventilation dives, foraging on surface-oriented prey). Dive durations and depths were reported in the behavior log with minimum and maximum estimates for each dive and surface record (depth sensor accuracy is ±1%) and we used the means of each parameter for analyses.

### Data processing

Tagged individuals were photo-identified following Baird et al. [36], assigning them to recognized populations (MHI, NWHI, or open-ocean) and to social groups derived from social network analyses (social clusters for MHI and NWHI individuals, and social components for open-ocean individuals; [41], CRC unpublished). Sex was determined genetically (if biopsy sampled; [39]), by morphological characteristics (e.g., head shape) if adequate photographs were available, or by the calf associations. If neither type of information were available, sex was assigned as “unknown”. Age class (calf, juvenile, sub-adult, adult) was determined based on field and photographic assessments and photo-identification catalog information [60]. For SPLASH deployments, fin base length was measured from photographs (using known tag dimensions, following [61]) and in some cases camera-based laser photogrammetry [62] to assess relative body size effects on dive behavior, as body size can vary substantially even within adults [63] and fin base length should be generally correlated with body length [61, 64]. Fin base length was presented as the mean of the measurements from all photographs of adequate quality.

After tag recovery, TDR data were processed using the manufacturer (Wildlife Computers) provided software *Minimum-Maximum-Mean* (Version 1.22) and *Zero-Offset-Correction* (Version 1.30) to correct for temperature-related drift in surface values. Those files were then processed with *Dive Analysis* (Version 4.08) to define and summarize statistics (maximum depth, duration, bottom time, ascent rate, descent rate) for each dive.

Behavior log data from the SPLASH satellite tags were examined for any indication of tag pressure transducer failures, drift (e.g., see [65, 66]), or other indicators of tag malfunctioning that could invalidate dive behavior data (e.g., see [67]). Our protocol and associated code are provided in a public repository [68].

Argos and Fastloc®-GPS location data from SPLASH tags were filtered in Movebank (Argos and GPS; [69]) and R (GPS only; [70]) to remove unrealistic locations based on speed and turn angles using species-specific settings specified in Kratofil et al. [71]. Filtered locations were subsequently fit to a continuous-time correlated random walk (CTCRW) model using the package *crawl* [72, 73] and used to estimate locations at behavior log event times. Any locations on land were rerouted around the 20-meter isobath with the *pathroutr* package [74].

### Data analysis

We used both TDR and SPLASH datasets to describe and visualize dive behavior at their respective scales and in relation to variables of interest. Formal statistical analysis was limited to the SPLASH behavior logs as this dataset includes more individuals and longer-duration deployments. Additionally, the TDR tags do not provide location data, and thus no assessment of spatial variables in relation to these data could be undertaken. A summary of datasets used for each broad analytical objective is provided in Table S1 (Additional File 2).

#### Dive metrics

Focal behavior metrics from the SPLASH tags included dive depth, dive duration, and dive rate, and these were each assessed in relation to spatial, temporal, and demographic variables. Dive depth can provide insight into different prey types that false killer whales may target, while dive duration may reflect foraging effort, such as the time spent searching for and pursuing prey during a given dive. Dive rate (i.e., the frequency of dives) may also be a measure of foraging effort. Dive rate was calculated as the total number of dives divided by the total duration of dive and surface periods. While we cannot explicitly infer foraging behavior from these dive metrics, we examine them in the context of foraging given that they are defined by dives 50+ m and 2+ min—extending to depth distributions of several known prey species (e.g., [75–77])—and thus are unlikely to reflect other behaviors (e.g., travelling, socializing). However, we acknowledge the possibility that shallower dives (e.g., ~ 50 m) could include other behaviors such as prey sharing. Median values of dive depth and duration were calculated for each individual, and individual medians were averaged for overall and group-level (e.g., demographic, temporal categories) means (following [78, 79]). We also provided the standard deviation (SD) and coefficient of variation (CV, expressed as percent) for group-level summaries. In addition to these descriptive summaries, statistical models were used to quantify relationships between dive metrics and spatial and temporal variables for SPLASH tag data (detailed in subsequent sections). Formal statistical analyses were not undertaken to examine dive behavior across demographic groups due to limited sample size.

#### Temporal and spatial variables

We assessed dive metrics by civil diel categories (i.e., dawn/dusk defined by −6/6 degrees above/below the horizon, day/night in between) to account for seasonal and geographic shifts in the timing of diel periods and allow comparisons with other studies. Sun angles were calculated using the *suntools* package [80]. Dive rate by diel category was calculated using the method described above, and extended surface periods spanning multiple diel categories were split by category using a custom R script to correctly calculate dive rate per category. Dive rates were also visualized across hours of the day (i.e., clock hour), and thus this same process was applied to split surface periods by clock hour when necessary. This approach ensures that dive rates are calculated without including any data gaps

While the abiotic and biotic characteristics vary across the ranges of the three populations, there are some spatial features common to all populations’ ranges (e.g., seafloor slope, chlorophyll-a levels, ocean current dynamics) and thus generalizable patterns could be inferred. Therefore, we constructed multivariate models relating dive metrics (i.e., dive depth, duration, hourly rate) to spatial and temporal predictors relevant to all populations and conducted separate univariate sub-analyses on additional spatial predictors relevant to each range-type (i.e., insular or open-ocean). For modeling hourly dive rate, the unit of observation was the number of dives per hour within each day of each tag deployment (e.g., 2010–10-28 01:00:00–01:59:59, 2010–10-28 02:00:00–02:59:59, etc.). For each individual tag deployment, hour-day units with less than 75% data coverage (i.e., summed duration of dives and surface periods in that hour < 0.75) were removed prior to modeling. Spatial predictors were not included in the hourly dive rate model as this would require fine-scale information on space use within each hour per day that is not typically available from satellite tags.

Candidate predictors included time-of-day, moon phase (in radians), day of the year, contemporaneous and 30-day lagged daily surface chlorophyll-a concentration (both log-10 transformed following [81]), daily mixed layer depth, daily sea surface temperature (SST), daily horizontal surface current magnitude, and seafloor slope. These dynamic oceanographic variables were selected because of their correlation with biological productivity and distribution of known false killer whale prey fish (e.g., yellowfin tuna or ʻahi, *Thunnus albacares*, [75, 82]). Time-of-day was modeled as a continuous variable as opposed to the categorical diel period to better assess non-linear relationships. Day of the year is confounded by individual variability as tags were deployed during different times of the year and durations were relatively short, and thus was not considered further. Moon phase was extracted using the *lunar* package [83]. Seafloor depth and slope were extracted from the 30 arc-second (~1 km resolution) GEBCO Grid^1^, and daily chlorophyll-a concentration (4 km horizontal resolution) and all other dynamic oceanographic variables (i.e., mixed layer thickness (hereafter mixed layer depth), SST, current magnitude; 8 km horizontal resolution) were obtained from the Copernicus Marine Data Store^2^^,^^3^. The chlorophyll-a product includes a “flag” variable to identify grid cells that overlap or partially overlap with land; dives within these cells were removed prior to modeling. Surface current magnitude was derived from zonal and meridional current velocity components following Woodworth et al. [84]. The 30-day lagged version of chlorophyll-a was derived using the value 30 days prior to that of the dive record. Seafloor depth was not included in multivariate models as this variable was not similarly relevant among the three populations (e.g., seafloor depth is not limiting to dive depth and is less variable for open-ocean false killer whales), but was explored for insular populations in a sub-analysis (details in the subsequent section). Spatial variables were extracted using the *stars* package [85].

Prior to analyses of dive behavior in relation to spatial features, we excluded dive records with positional error ellipse estimates greater than 4 km so that retained dives would not exceed the spatial resolution of the dynamic oceanographic variables of interest, or drastically influence inference on static variables (e.g., slope). To assess whether this data restriction obscured any temporal patterns in dive behavior, we also fit all models with temporal predictors only, using the full dive records.

#### Generalized additive mixed effects models

Dive metrics were modeled in relation to several temporal and spatial variables using generalized additive mixed effects models (GAMMs) to allow for non-linear trends. GAMMs were fitted using the *mgcv* R package [86] with the restricted maximum likelihood method. Response variables dive depth and dive duration were each modeled with a Gamma distribution and log link function. Hourly dive rate was modeled as dive count per hour with a negative binomial distribution and log link function, and an offset for log hours of behavior data per hour to effectively model dive rate. Correlation among predictor variables was assessed prior to modeling; if predictors were highly correlated (Pearson’s correlation > 0.5), only one of the predictors was retained in the model. Time-of-day and moon phase were modeled with smooth cyclic cubic regression splines (bs = “cc”), while all spatial predictors were modeled with thin plate regression splines (bs = “tp”). For each smooth term, we examined the model outcomes across different basis dimension sizes (k = 5 … 20) and ultimately constrained this value to five (k = 5) to balance model fit and interpretability [86]. Variable selection was conducted with a shrinkage approach that penalizes non-significant variables to zero [87]. We evaluated the importance of individual-level variation by fitting two models with different random effects structures: (1) a model with random intercepts for tag ID (i.e., PcTag + tag deployment number; bs = “re”); and (2) a model with random factor smooths for tag ID (bs = “fs”) on time-of-day to allow for individual-level variation in diel trends [88]; the same shrinkage approach described above was applied to both models. An individual-level factor smooth term of tag ID with other predictors was not evaluated as not all tagged whales experienced the same range of predictor values (in contrast to time-of-day). We used conditional Akaike’s Information Criterion (AIC) [89] and percent deviance explained to determine the best fit between these two random effects structures. Models were validated by visually inspecting residuals and through the gam.check() function (i.e., to assess adequacy of basis dimension size), and model performance was assessed through the percentage of the deviance explained. Following recent statistical recommendations [90], we report covariate-specific p-values quantified in the GAMMs to aid interpretation, but these were not used as the sole basis of covariate significance. We additionally calculated covariate-specific contributions to the total percent deviance explained using the *gam.hp* package [91]; currently, there is no functionality to obtain these values from models with offsets, so these were not determined for the hourly dive rate model. Model prediction plots were created using the *marginaleffects* R package [92]. For these prediction plots, covariate-specific relationships were conditioned upon the mean value of all other covariates in the model, and thus represent the functional relationship for a “typical” individual [92].

#### Near-seafloor diving behavior

Insular false killer whales often use shallow, nearshore waters where the seafloor is accessible, and thus they have the potential to target prey near the seafloor. To explore this further, we needed to discern whether dives near the seafloor were an artifact of tag positional uncertainty or if the animals were truly diving near the seafloor. We first generated 20 imputations from the fitted CTCRW models for each individual, such that we obtained 20 possible routes that each individual could have taken given the parameterized movement model (e.g., CTCRW process, location error, time between locations, etc., see [93]). Positions were then estimated for each dive record from each of the 20 imputations using the method described previously, such that each dive record had 20 plausible geographic locations. Seafloor depths were extracted for each of the 20 dive locations using higher resolution bathymetry grids available for the ranges of insular false killer whales (MHI: 50-meter MHI Multibeam Bathymetry Grid^4^; NWHI: 60-meter NWHI Falkor Bathymetry grid^5^). We narrowed the subset to dives within the spatial uncertainty restriction criteria applied for the modeling (described above), then calculated the standard deviation and maximum seafloor depth values across the 20 imputations for each dive record. To focus on dives with reasonable spatial precision, probable seafloor dives were isolated by removing dives that had a standard deviation of seafloor depth values greater than 100 m. We then subset dives with a maximum depth difference (i.e., maximum seafloor depth minus dive depth) of ± 100 m or less to narrow down a window of the water column where dives could be close to the seafloor while accounting for the fact that seafloor depth can vary substantially around some habitat features. These were further examined visually and quantitatively (e.g., through assessing distributions of seafloor depth between imputed dives and estimated dive locations, the ratio of dive depth to seafloor depth) to assess the likelihood of dives occurring to or close to the seafloor.

## Results

### Tag deployment summary

Five individuals were tagged with TDR tags between 1999 and 2008, and sixteen individuals were tagged with SPLASH satellite tags between 2010 and 2025 (Table 1). Based on photo-identification, all tags were deployed on different individuals. All TDR deployments and eight SPLASH satellite tag deployments were on individuals from the MHI population, five SPLASH tags were on individuals from the NWHI population, and the remaining three SPLASH tags were on open-ocean false killer whales. Within the MHI population, tagged individuals represented three of the four recognized social clusters (Table 1; [41]). For the NWHI population, tagged individuals represented three of seven identified clusters (CRC unpublished). For the open-ocean population, there was one pair of tagged individuals that belong to the same component in the social network, and one individual from a separate component (Table 1). Sex was known for 14 individuals (TDR: three males, one female; SPLASH: seven males, three females, Table 1). All but two of the SPLASH tag deployments were on adults (the remaining deployments were on a sub-adult and juvenile), while only two of the five TDR deployments were on adults (Table 1).

**Table 1:** False killer whale dive behavior tag deployment summary

Tag ID	Tag type	Population–cluster/component	Age class	Sex	Fin base length mean ± SD (cm)		Deployment date	Deployment locality	Behavior data available (% coverage)
PcTDR01	Mk6 TDR	MHI – C3	A	U		-	29-Mar-99	Maui Nui	1.1 h
PcTDR02	Mk6 TDR	MHI – C4	A	F^2^		-	17-Nov-99	Maui Nui	13.4 h
PcTDR03	Mk6 TDR	MHI – C1	J	M^1^		-	28-Feb-01	Maui Nui	3.6 h
PcTDR04	Mk6 TDR	MHI – C3	SA	M^1^		-	06-Oct-04	Hawaiʻi Island	28.9 h
PcTDR05	Mk9 TDR	MHI – C1	J	M^1^		-	16-Jul-08	Hawaiʻi Island	7.3 h
*Total*									54.3 h
PcTag026	SPLASH10	MHI – C3	A	M^1^		68.0 (0.1)	15-Oct-10	Oʻahu	7.0 d (64.1)
PcTag028	SPLASH10	MHI – C1	SA	U		57.3 (0.4)	22-Oct-10	Oʻahu	15.7 d (40.3)
PcTag030	SPLASH10	MHI – C3	A	U		55.2 (NA)	11-Dec-10	Hawaiʻi Island	20.0 d (66.8)
PcTag032	SPLASH10	MHI – C3	A	M^1^		65.8 (0.5)	11-Dec-10	Hawaiʻi Island	20.1 d (77.1)
PcTag055	SPLASH10-F	MHI – C4	A	M^1^		68.0 (NA)	09-Mar-17	Maui Nui	6.5 d (78.4)
PcTag074	SPLASH10-F	MHI – C3	A	U		56.5 (0.2)	08-Aug-21	Kauaʻi	9.2 d (95.7)
PcTag095	SPLASH10-F	MHI – C1	J	F^1^		NA (NA)	20-Jan-25	Hawaiʻi Island	26.2 d (84.7)
PcTag099	SPLASH10-F	MHI – C1	A	M^1^		67.3 (NA)	13-Jul-25	Hawaiʻi Island	25.7 d (86.7)
*Total*									130.5 d
PcTag035	SPLASH10	NWHI – C5	A	M^1^		66.7 (NA)	13-Jun-12	Kauaʻi	6.6 d (100.0)
PcTag037	SPLASH10	NWHI – C4	A	M^2^		66.8 (0.2)	26-Jul-13	Kauaʻi	10.2 d (67.0)
PcTag049	SPLASH10	NWHI – C2	A	U		57.9 (NA)	06-Sep-15	Kauaʻi	14.5 d (98.6)
PcTag096	SPLASH10-F	NWHI – C4	A	U		55.7 (NA)	17-Feb-25	Kauaʻi	3.8 d (59.4)
PcTag097	SPLASH10-F	NWHI – C4	A	U		56.6 (NA)	17-Feb-25	Kauaʻi	7.1 d (94.0)
*Total*									42.2 d
PcTag090	SPLASH10-F	Open-ocean – MC1	A	F^1^		56.8 (NA)	31-Oct-23	Hawaiʻi Island	16.3 d (99.6)
PcTag092	SPLASH10-F	Open-ocean – MC1	A	F^1^		56.7 (NA)	31-Oct-23	Hawaiʻi Island	3.8 d (45.4)
PcTagP09	SPLASH10-F	Open-ocean – IC2	A	M^1^		65.9 (0.4)	16-May-24	E. Johnston Atoll	8.4 d (69.1)
*Total*									28.4 d

Social network cluster for MHI individuals was from Mahaffy et al. (2023). Sex was either genetically determined from a biopsy sample^1^, inferred from presence of calf or morphological characteristics^2^, or unknown if neither source of information were available. Percent behavior data coverage for SPLASH tags represents the summed duration of behavior log records out of the total time of behavior log data transmission (i.e., first message to last message), and thus provides an indication of gaps in transmissions. Individuals with multiple photos for dorsal fin base length measurements have an associated standard deviation (SD) with the mean of measurements. There is no dorsal fin base measurement for PcTag095 due to a lack of sufficient quality photographs for measurements. MHI = Main Hawaiian Islands; C = Cluster; NWHI = Northwestern Hawaiian Islands; MC = Main Component; IC = Isolated Component; A = adult; SA = sub-adult; J = juvenile; F = female; *M* = male; U = unknown. Maui Nui includes the islands of Maui, Molokaʻi, Lānaʻi, and Kahoʻolawe

### Short-term, fine-scale dive behavior

TDR attachment duration ranged from 1.1 to 28.9 h for a combined 54.3 h across all deployments (Table 1). Of the 54.3 h of data, there were 1.0 h during dawn, 32.2 h during the day, 2.9 h during dusk, and 18.2 h during night. TDR-tagged whales spent an overall mean of 70.5% of their time in the top 10 m of the water column (range = 49.5–90.5%), with almost all their time spent in the top 50 m of the water column (Table S2, Figure S1, Additional File 2). Out of all 716 dives to 10 m or deeper, dives were relatively shallow with an overall mean dive depth of 15 m (range of individual median depths = 11–19 m; SD = 3 m; CV = 21.8) and duration of 1.5 min (range of individual median durations = 0.9–1.9 min; SD = 0.4 min; CV = 26.7). The longest and deepest dive was 234 m deep and 12.0 min long; however, based on the dive profile at this time and the depth range of the tag sensor, this individual (PcTDR04) had maxed out the depth sensor for this tag. Thus, while the duration of this dive is accurate, the true dive depth was deeper than 234 m. Dives exceeding 230 m depth from this individual were thus excluded from further analyses. The deepest dive after this exclusion was 210 m (5.4 min long). No comparisons were made across demographic groups (i.e., social cluster, age, sex) due to limited tag deployments by groups and highly variable amounts of behavior data among individuals (Table 1).

For the three tags (PcTDR02, PcTDR04, and PcTDR05) that had data available across different times of day, mean time spent in water depths greater than 50 m was low during the day (1.0%; SD = 0.4%; CV = 40.1) and night (4.0%; SD = 4.9%; CV = 121.1). Overall mean dive depths during the day (17.7 m; SD = 1.5 m; CV = 8.6) and night (17.7 m; SD = 4.9 m; CV = 27.9) were similar and both deeper than those during dusk (13.3 m; SD = 2.5 m; CV = 18.9; Figure S2, Additional File 2). Trends in dive durations followed a similar pattern, with overall mean durations during the day (1.6 min; SD = 0.3 min; CV = 17.0) and night (1.9 min; SD = 1.1 min; CV = 55.2) being longer than at dusk (1.2 min; SD = 0.1 min; CV = 10.0; Figure S2). Descent rates were higher than ascent rates, and this trend was consistent across diel periods (Figure S2, Additional File 2). There were 37 dives with greater than 2 m/s descent rates. The maximum descent rate recorded was 5.8 m/s and associated with a daytime dive to 30 m. Mean relative velocity readings for PcTDR02 were highest at night (0.61), followed by day (0.47) and dusk (0.31), but the maximum was recorded during the day (6.8). Mean relative velocity readings were higher overall for PcTDR04, and throughout the diel period, were highest during dawn and day (1.3, 1.3, respectively) compared to dusk (0.84) and night (0.40); the maximum relative velocity for this individual (6.6) was also recorded during the day.

**Fig. 1:**
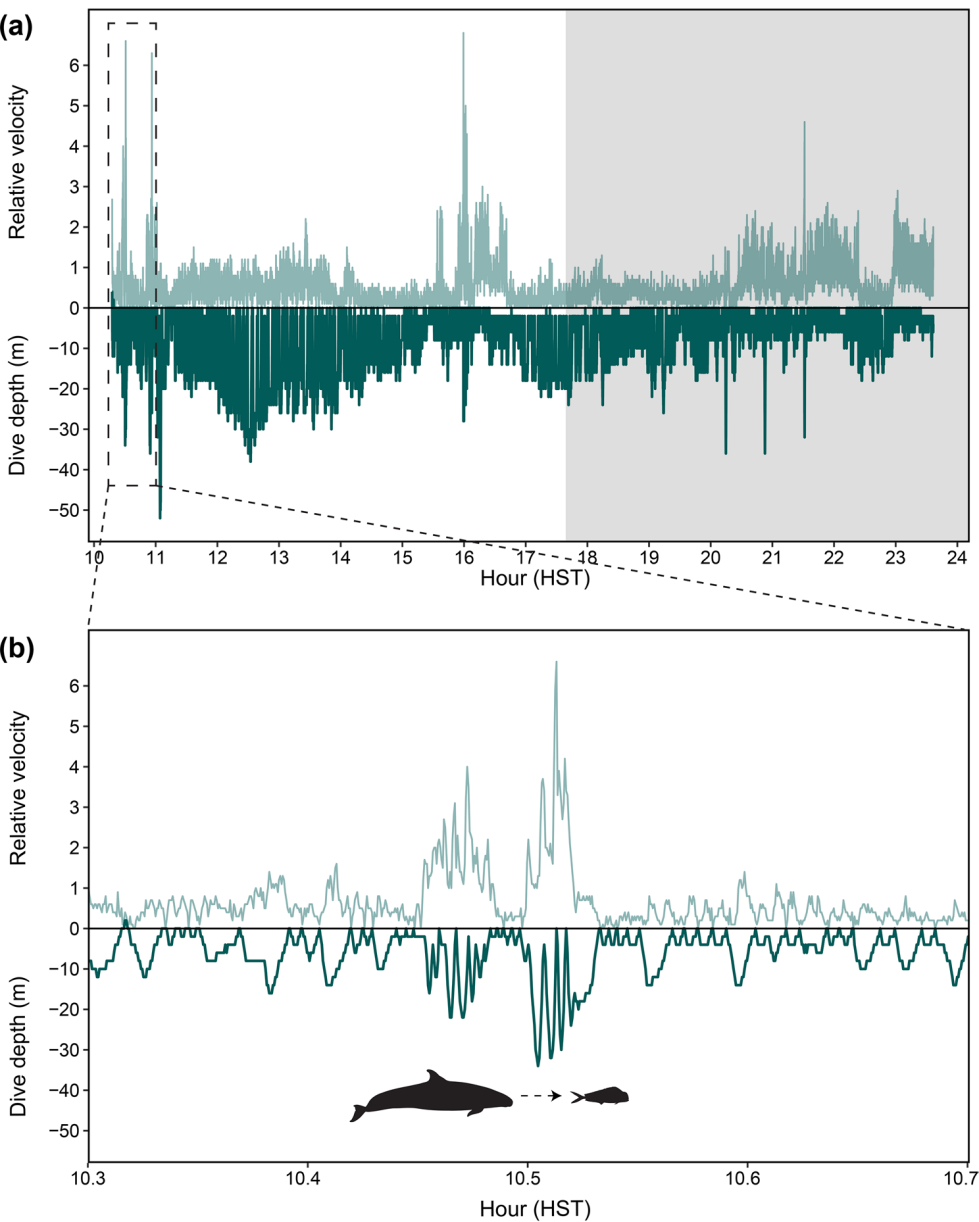
Dive depth and relative velocity profile from false killer whale PCTDR02 tagged in November 1999. Dives are represented by dark green lines and swim speed by light green lines. Night periods are indicated by gray shading and hours are in Hawaiian Standard Time (HST). (**a**) Dive and velocity measurements over the entire deployment period; (**b**) Dive and velocity profiles during a short period of time when the animal was observed chasing a mahimahi (indicated by the mahimahi icon)

One tagged individual (PcTDR02) was observed chasing a mahimahi shortly after tag deployment and dove within the top 50 m of the water column during this pursuit (Fig. 1). There were two bouts of increased velocity during this prey chase, which reached maximums of 4.0 and 6.6 (uncalibrated units), respectively. No feeding events were observed post-tagging for the remaining individuals, but tagged animals were not systematically followed post-tagging to record such behavioral information due to other research priorities.

### Long-term, coarse-scale dive behavior

Tag transmission durations of SPLASH tags ranged between 8.7 and 47.6 days (median = 20.1 d). Two deployments likely failed due to low battery voltage, while the others presumably failed due to the tag becoming detached. Coverage of behavioral data (i.e., proportion of data obtained out of the total data transmission period) was variable, ranging from 40.3 to 100% (Table 1). Excluding gaps in the behavior log data, there were 201.1 days of behavior data combined across all deployments (median = 9.7 d, range = 3.8–26.2 d; Table 1). By diel period, there were 7.3 days of data during dawn, 93.0 days during day, 8.1 days during dusk, and 92.7 days during night. There were no indications of pressure transducer failures for any of the tags.

**Fig. 2:**
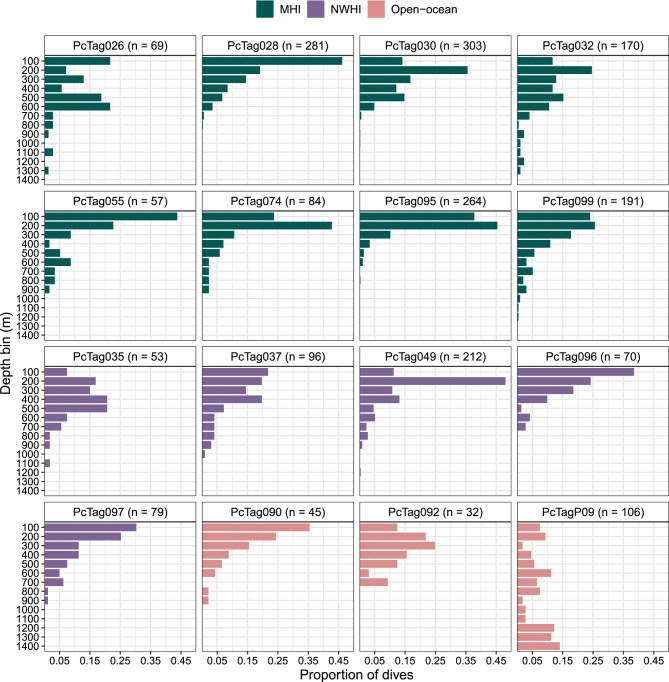
Proportions of dives by depth bin for each SPLASH-tagged false killer whale. The first depth bin labeled as “100” on the y-axis represents dives between 50 and 100 meters deep; the subsequent bin (“200”) therefore represents dives 101–200 meters deep. MHI = main Hawaiian Islands; NWHI = Northwestern Hawaiian Islands. The number of dives for each tag are shown in plot headers

All tagged whales spent most of their time in the top 50 m of the water column (range = 89.5–99.0% time at “surface”; Table S3, Additional File 2). A total of 2112 standardized dives (50 m or deeper, 2 min or longer) were recorded, ranging from 32 to 303 dives per individual (Table S3, Additional File 2). For dives 50 m deep or greater, the overall mean dive depth was 246 m (range of individual median depths = 112–744 m, SD = 164 m, CV = 66.5.9) and overall mean dive duration was 5.8 min (range of individual median durations = 3.8–13.4 min, SD = 2.2 min, CV = 37.4; Table S3, Additional File 2). The deepest dive recorded was 1424 m from an open-ocean false killer whale; this was also the longest dive recorded (19.1 min; Table S3, Additional File 2). This individual (PcTagP09) had visually different diving behavior from all other tagged whales, having higher proportions of deeper (greater than 750 m) dives than observed in other whales (Fig. 2). The deepest dive recorded in the MHI population was 1272 m (14.7 min) and the longest dive was 18.6 min (1,264 m; Table S3, Additional File 2). The deepest dive for NWHI individuals was also the longest dive, recorded at 1104 m and 18.2 min. Dive durations were positively correlated with dive depths (Spearman’s rank correlation coefficient = 0.72); there were no apparent breaks that would indicate the existence of distinct dive types within the range of dive depths and durations we standardized herein (i.e., ≥ 50 m deep, ≥ 2 min long; Figure S3, Additional File 2). Mean dive rate was 0.45 dives per hour (SD = 0.17, CV = 37.8) and ranged from 0.12 dives per hour to 0.75 dives per hour (Table S3, Additional File 2). The mean time spent in the top 50 m of the water column between dives greater than 50 m was 51.0 min (range of individual medians = 19.3–86.5 min, SD = 21.1 min, CV = 41.1). Two individuals remained in the top 50 m for multiple days: one individual from the MHI population (max surface period duration = 3.1 d) and one individual from the open-ocean population (max surface period duration = 5.1 d).

#### Demographic comparisons

In the following results, descriptive summaries reflect those of the sample (i.e., data from SPLASH-tagged whales presented in this study), as limited group-specific sample sizes preclude generalizations on the respective between-group comparisons. Sample population-level summary statistics indicate that open-ocean false killer whales dove deeper (group mean = 390 m, SD = 317 m, CV = 81.2) and longer (group mean = 8.0 min, SD = 4.8 min, CV = 0.60) than NWHI (group means = 220 m, 5.5 min; SDs = 99 m, 0.7 min; CVs = 44.9, 12.0, respectively) and MHI false killer whales (group means = 208 m, 5.2 min; SDs = 110 m, 1.0 min; CVs = 53.1, 19.7, respectively), however this was driven by the one open-ocean individual with the greatest dive depths (PcTagP09; open-ocean group means with PcTagP09 excluded = 214 m, 5.3 min; SDs = 116 m, 1.3 min; CVs = 54.3, 23.8, respectively). Mean dive rates were lower in open-ocean false killer whales compared to MHI and NWHI false killer whales (group means = 0.33, 0.45, 0.51 dives/h, respectively), but variability within this metric was highest for open-ocean false killer whales compared to the other two populations (SDs = 0.21, 0.15, 0.17 dives/h; CVs = 62.2, 33.9, 33.7, respectively). Males dove deeper (group mean = 350 m, SD = 200 m, CV = 57.1) and longer (group mean = 6.8 min, SD = 3.0 min, CV = 44.1) than females (group means = 182 m, 4.9 min; SDs = 98 m, 1.2 min; CVs = 54.0, 24.2, respectively) and those of unknown sex (group means = 156 m, 5.2 min, SDs = 32 m, 0.9 min; CVs = 20.6, 17.5, respectively), although within-group variation was apparent and sample size varied by group (Table 1; Figure S2, Additional File 2). Excluding PcTagP09, the group mean for males was still deeper (285 m, SD = 109 m, CV = 38.4) than that for females and unknown sex individuals (with the sample size caveats noted above), but mean dive duration (group mean = 5.7 min, SD = 0.7 min, CV = 12.4) was more comparable to females and those of unknown sex. Dive rates were highest in individuals of unknown sex (mean = 0.60, SD = 0.15, CV = 25.3), followed by males (mean = 0.38, SD = 0.07, CV = 18.5), and females (mean = 0.29, SD = 0.16, CV = 54.1), although variation was highest in females (Figure S4, Additional File 2). Between-social cluster comparisons were not made for MHI individuals due to limited representation among clusters (Cluster 1, *n* = 3; Cluster 3, *n* = 4; Cluster 4, *n* = 1; Table 1). Within Cluster 3, the coefficient of variation for median dive depth was moderate (CV = 44.3; SD = 121 m) and low for median dive duration (CV = 24.6; SD = 1.3 min) and dive rate (CV = 28.8; SD = 0.13 dives/h). No age class comparisons were made as all but two SPLASH-tagged individual were adults (Table 1), however we assessed the potential influence of relative body size using fin base length as a proxy. Fin base length ranged from 55.2 cm to 68.0 cm; known males had fin base lengths greater than 65 cm and known females were less than 60 cm; individuals of unknown sex had some of the smallest fin base lengths (Table 1). There was a positive correlation between fin base length and median dive depth (Spearman’s rank correlation = 0.39) and median dive duration (Spearman’s rank correlation = 0.38). Five of six individuals that dove greater than 1000 m deep had fin base lengths greater than 65 cm, but some individuals with longer fin bases did not dive as deep (Table 1; Table S3, Additional File 2).

### Spatial and temporal patterns in dive behavior

**Fig. 3:**
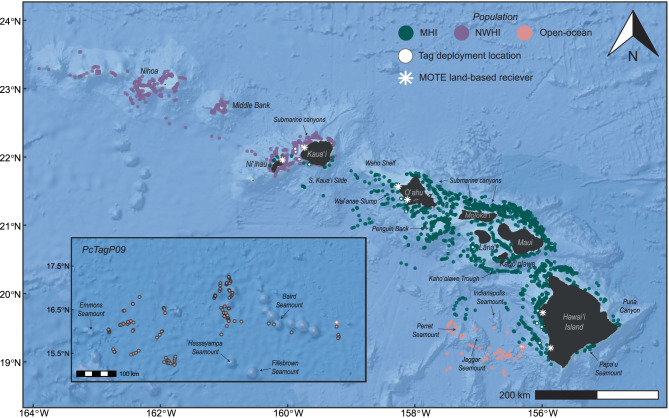
Estimated dive locations from sixteen SPLASH-tagged false killer whales in the study area. Dives (≥50 m, ≥ 2 min) are represented by circles. Note that the two deployment locations for open-ocean false killer whales off west Hawaiʻi Island overlap. The inset map shows dive locations for PcTagP09 which occurred much farther offshore from the islands. Select topographic features are labeled

**Fig. 4:**
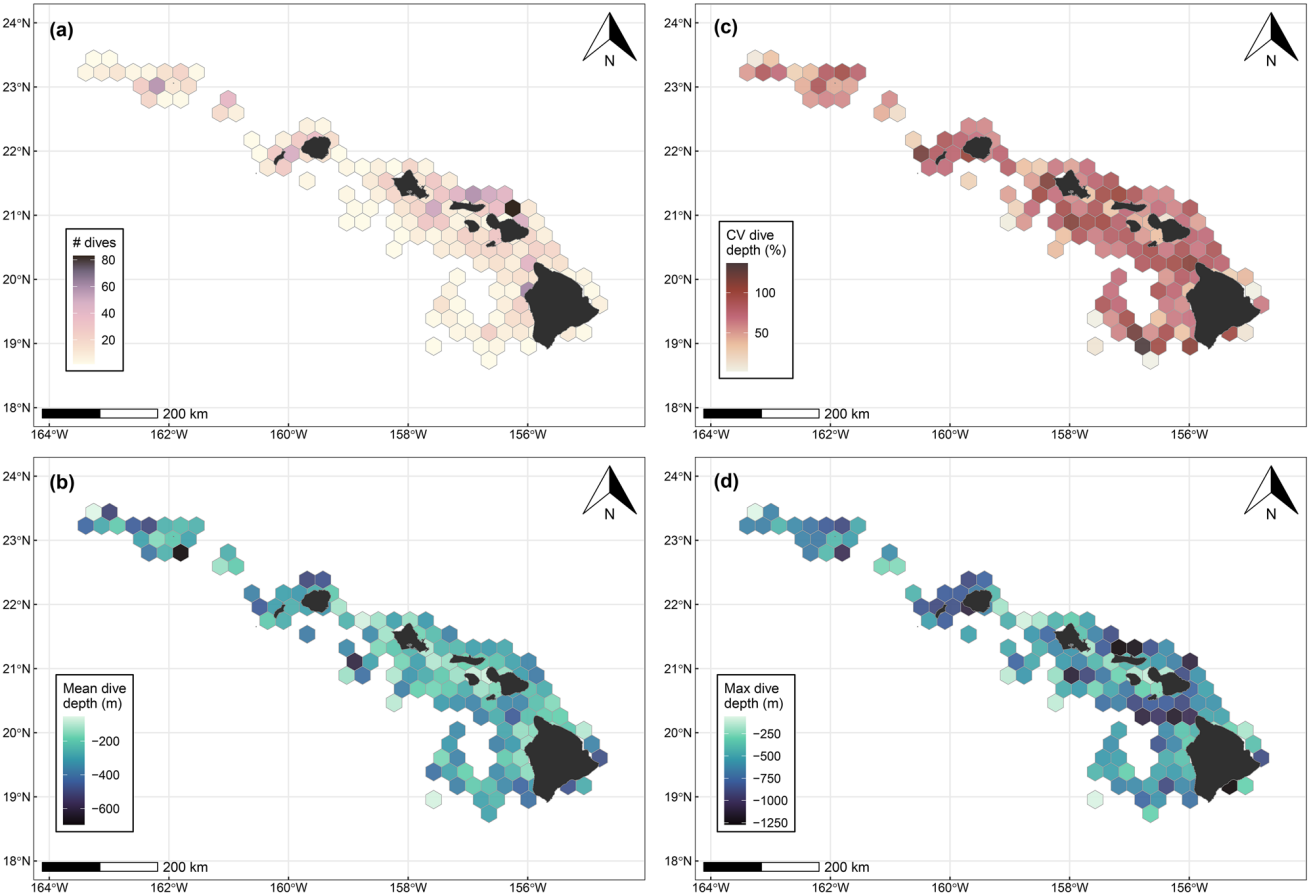
Gridded spatial distribution of dives from SPLASH-tagged false killer whales. (**a**) Number of dives; (**b**) Mean dive depth; (**c**) Coefficient of variation (CV) in dive depth; and (**d**) Maximum dive depth. Hexagon cell size (for those not intersecting with land) is 750 km^2^, and cells with only one dive were excluded from these summaries. PcTagP09 was not included in these visual summaries because it was the only individual that occurred in its geographic range, precluding among-individual spatial summaries of dive metrics

Dives occurred in a variety of habitats with differences among populations (Fig. 3). MHI false killer whales dove in both nearshore and offshore environments, with the most dives recorded off the windward side of Maui Nui, around Oʻahu, and on Penguin Bank, all areas where the tagged individuals spent most of their time (Figure S5, Additional File 2). These areas include island shelf/slope transitions of varying degrees and submarine canyons (north of Molokaʻi; Fig. 3). NWHI false killer whales also dove primarily in nearshore habitats, with most dives concentrated around Kauaʻi, Niʻihau, Middle Bank, and Nihoa (Fig. 3), where tagged animals spent most of their time (Figure S5, Additional File 2). The 2023 tagged pelagic false killer whales dove around the Geologists Seamounts west of Hawaiʻi Island (e.g., Perret, Cook, Jaggar Seamounts), and the 2024 individual dove around chains of seamounts and abyssal plains farther from the islands (Fig. 3; Figure S5, Additional File 2). While mean dive depth was typically shallower in island shelf and slope habitats, there was also high variability in dive depth within these habitats (CV of dive depth > 50%), including both shallow and very deep dives (Fig. 4).

**Fig. 5:**
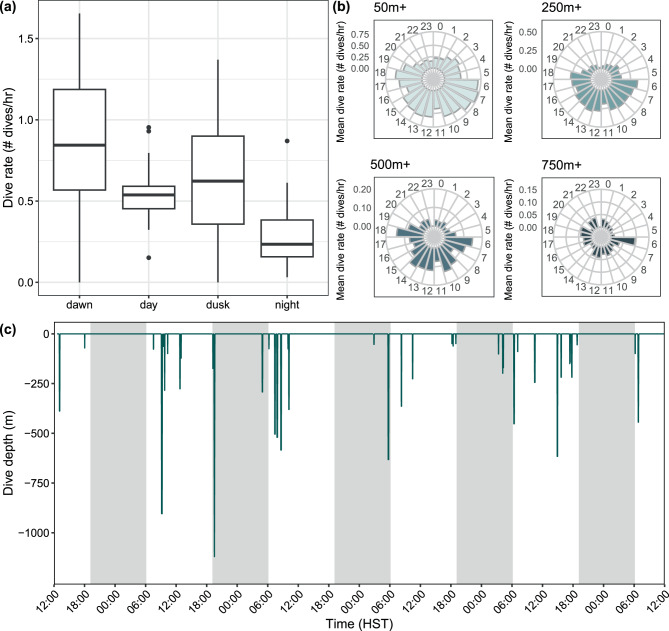
Grand mean dive rates throughout the diel cycle and across dive depth bins. Dive rates (# dives per hour; dives ≥ 50 m and ≥ 2 min) for (**a**) Each diel category and (**b**) Hourly, across different depth bins. A single value per individual and diel category was used for (**a**) and overall mean values were used for (**b**). Panel (**c**) Shows the dive depth profile over five days for one tagged whale (PcTag099), with nighttime represented by the grey shaded rectangles (HST = Hawaiian Standard Time). Boxplots: the middle line in each box represents the median, the lower and upper extents of the box the first and third quartiles (respectively), the lower and upper whiskers the minimum and maximum (quartiles ±1.5*interquartile range), and any values outside of the box/whiskers are shown as points

Univariate summaries of dive metrics by diel period indicate that mean dive depth and duration were similar across diel categories (Table 2). In contrast, dive rates were highest at dawn and dusk, intermediate during the day, and lowest at night (Table 2, Fig. 5). This trend persisted across most depth bins, but became less apparent for deeper dives (500 m+, 750 m+, Fig. 5). While dives greater than 750 m were generally infrequent, they occurred most often early in the morning and otherwise at relatively similar rates within the diel cycle (Fig. 5). The average proportion of square-, u-, and v-shaped dives were similar throughout the diel cycle, with slightly higher proportions of u-shaped dives during dusk and v-shaped dives during night (8–10% more; Table 2). However, there was high variability in nearly all of these univariate summaries (Table 2). Dive metrics by shape and diel category were highly comparable to the patterns observed in dive metrics across diel categories irrespective of dive shape (Figure S6, Additional File 2).

**Table 2:** Summary of dives metrics from SPLASH-tagged false killer whales by diel category

	Mean ± SD (CV)						
Diel category	# dives/total	Dive depth (m)	Dive duration (min)	Rate (# dives ≥50 m/h)	% Square-shaped dives	% U-shaped dives	% V-shaped dives
Dawn	10 ± 7 (69.7)/155	273 ± 186 (68.2)	6.3 ± 3.1 (49.3)	0.92 ± 0.39 (42.6)	34.5 ± 28.7 (83.1)	52.7 ± 26.7 (50.7)	12.8 ± 12.6 (98.2)
Day	74 ± 51 (68.7)/1179	270 ± 162 (59.9)	6.0 ± 2.3 (39.2)	0.55 ± 0.21 (37.5)	27.1 ± 20.2 (74.6)	63.1 ± 18.1 (28.6)	9.8 ± 5.3 (53.9)
Dusk	10 ± 7 (66.5)/137	292 ± 171 (58.3)	6.3 ± 2.5 (40.5)	0.72 ± 0.32 (45.2)	19.7 ± 27.5 (139.6)	70.9 ± 29.1 (41.0)	9.4 ± 12.5 (133.1)
Night	40 ± 38 (95.4)/641	216 ± 235 (109.1)	5.6 ± 2.4 (43.1)	0.29 ± 0.21 (71.6)	25.6 ± 22.6 (88.3)	56.0 ± 22.6 (40.4)	18.3 ± 10.6 (58.0)

Grand mean (mean of medians or proportions) ± the standard deviation (SD) and coefficient of variation (CV, expressed as %) of dive metrics from SPLASH-tagged false killer whales by diel category. Dives were considered those 50 m or deeper and 2 min or longer. The number of dives column includes the grand mean, SD, and CV across individuals and the total number of dives for the diel category. Dive shapes are determined by different proportions of bottom time within dives

#### Spatiotemporal drivers of dive metrics

A total of 1775 dives were available for the full models (i.e., both spatial and temporal predictors) after removing dives with positional uncertainty greater than 4 km (337 dives) and those with missing covariate values (19 dives). Contemporaneous daily chlorophyll-a concentration and SST were correlated with 30-day lagged chlorophyll-a concentration (Pearson’s correlations = 0.7, 0.5, respectively) and thus we selected the latter variable (i.e., 30-day lagged chlorophyll-a) for subsequent modeling and excluded the former two. Correlations among all remaining pairs of predictor variables were low (Pearson’s correlations < 0.5), and thus these variables were retained in the models. The GAMM models indicated that individual variation was notable across all models. The best fit model for each dive metric included the random factor smooth structure for time-of-day and individual tag ID, with the percent of deviance explained increasing by 1.0–4.6% compared to the random intercept model (Table S4, Additional File 2). Therefore, we only report results on the models including the random factor smooth term throughout the rest of this section.

**Fig. 6:**
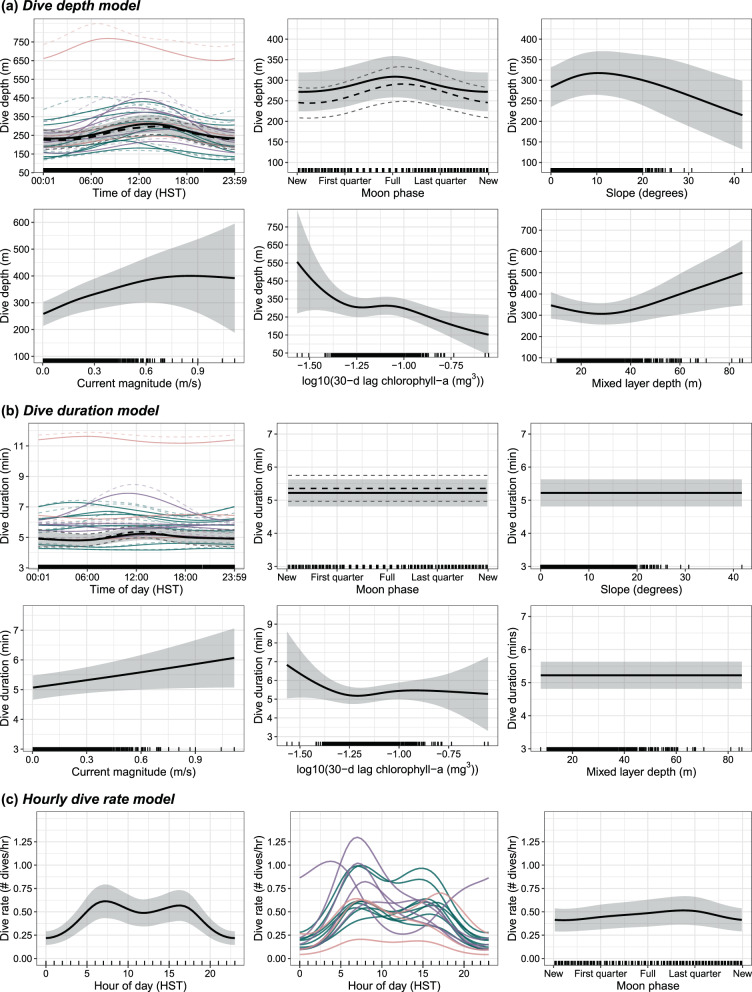
Predicted relationships from generalized additive mixed effects models. (**a**) Dive depth; (**b**) Dive duration; and (**c**) Hourly dive rate in relation to spatial and temporal predictors (temporal only for (**c**)). The mean predicted functional response curve for each covariate is conditional upon the mean value for all other covariates, and is represented by the solid black line and associated 95% confidence intervals are represented by the shaded gray ribbon. Panels with colored lines show the random smooths for each individual and time of day, colored by population assignment (green = MHI, purple = NWHI, pink = open-ocean). Observed predictor values are shown as vertical black ticks along the x-axis. Dashed lines in time of day and moon phase panels in (**a**) and (**b**) represent the predicted response curves for those predictors in the model fit with the full dataset (i.e., no data restriction applied) and only temporal predictors

The dive depth model (32.2% deviance explained) indicated that tagged false killer whales generally dove deeper during daylight hours with a peak around mid-day (Table 3, Fig. 6a). However, this relationship varied significantly across individuals, with individual-level random effects contributing 25.6% of the total deviance explained by the model (Table 3, Fig. 6a). Most tagged individuals had little to no trend in dive depth across time-of-day (Fig. 6a). The individual with the most apparent diel trend in diving depth (PcTag049) was from the NWHI population, reflecting the overall trend of diving deeper in the middle of the day. Two individuals from the MHI population appeared to have slightly deeper dives at dawn (PcTag028) or dusk (PcTag074; Fig. 6a). Chlorophyll-a concentration (30-d lag) had the next highest contribution to the model’s deviance explained (3.3%), where dives became shallower from low to intermediate chlorophyll-a concentrations, were no affected at intermediate values, followed by another decrease in dive depth with higher concentrations (Fig. 6a). The model also indicated that whales dove deepest during full moons, however the predicted effect was relatively weak (0.08% deviance explained; Table 3, Fig. 6a). Dive depths varied non-linearly with current magnitude (2.0% deviance explained; increase in depth with magnitude but asymptotes at higher values) and mixed layer depth (1.0% deviance explained), with no change in dive depth up to approximately 30 meters mixed layer depth, followed by an increase in dive depth with deeper mixed layers (Fig. 6a). Seafloor slope had a minimal effect on dive depths (0.2% deviance explained), with little change in dive depth with slope until higher slope values, where depth slightly decreased (Fig. 6a). However, this decreasing trend was driven by few observations with higher slopes (Fig. 6a).

**Table 3:** Generalized additive mixed effects model outputs for dive behavior metrics

Model	Covariate	EDF	F-value	p-value	Deviance explained (%) by covariate
Dive depth	Time-of-day (HST)	2.260	4.683	< 0.001	0.01
	Moon phase	1.739	1.769	0.032	0.08
	Slope	1.775	1.606	0.020	0.24
	Current magnitude	2.012	5.382	< 0.001	2.03
	Chlorophyll-a (30-d lag)	3.153	3.233	0.003	3.28
	Mixed layer depth	2.052	2.962	< 0.001	1.00
	Random: time-of-day ×ID	31.973	2.712	< 0.001	25.56
	Total	-	-	-	32.21
Dive duration	Time-of-day (HST)	0.207	0.076	0.129	0.05
	Moon phase	0.006	0.002	0.465	0.04
	Slope	0.001	0.000	0.712	0.34
	Current magnitude	0.830	1.186	0.015	1.08
	Chlorophyll-a (30-d lag)	2.693	1.676	0.045	1.84
	Mixed layer depth	0.004	0.001	0.520	0.25
	Random: time-of-day ×ID	27.890	3.387	< 0.001	24.4
	Total	-	-	-	28.00
Hourly dive rate	Hour-of-day (HST)	2.914		< 0.001	-
	Moon phase	2.023		0.011	-
	Random: hour-of-day ×ID	45.241		< 0.001	-
	Total	-			14.70

Dives with positional uncertainty of 4 km and greater were removed prior to analyses for dive depth and duration models that include both spatial and temporal predictors. All dives were used for the hourly dive rate model. The percent deviance explained by covariates for the hourly dive rate model are not included, as there is currently no functionality to obtain this metric from a model that includes an offset term. EDF = estimated degrees of freedom

The model for dive duration (28.0% deviance explained) showed similar trends for time-of-day, where dives were generally longer at mid-day with significant among-individual variation (24.4% deviance explained; Table 3, Fig. 6b). The individual-level trends observed in the dive duration model largely align with those in the dive depth model, albeit at slightly lower magnitude than observed in dive depths (Figs. 6a, b). Similar to the dive depth model, chlorophyll-a concentration (30-d lag) had the second highest contribution to the overall deviance explained (1.8%), although the predicted relationship was slightly different for dive duration (Fig. 6b). Dives were the longest at low chlorophyll-a concentrations and decreased at intermediate concentrations; however, this final portion of the predicted curve was driven by only a few observations (Fig. 6b). Current magnitude contributed a similar proportion of deviance explained to the model (1.1%) with an estimated positive relationship between dive duration with increasing current magnitude (Fig. 6b). The remaining covariates (moon phase, slope, mixed layer depth) had no estimated effect on dive duration (Table 3, Fig. 6b).

The trends observed in the full models with the restricted dataset were nearly identical to those in the temporal predictors-only models fit with the complete dataset (*n* = 2112 dives; Figs. 6a, 6b; Table S5, Additional File 2). Statistically, the dive duration model with the restricted dataset and all predictors estimated no effect of time-of-day (*p* = 0.129; Table 3, Fig. 6b), while the temporal predictors-only model estimated a significant relationship (*p* = 0.017; Fig. 6b; Table S5, Additional File 2). Visually, the predicted effects of this relationship were highly comparable between the two datasets (Fig. 6b). Additionally, models without data from PcTagP09 (the individual with the most different dive behavior) showed the same trends as the models with all individuals (Table S6, Additional File 2).

Hourly dive rates were highest between approximately 06:00 and 18:00 (Hawaiian Standard Time) and the model estimated a bimodal distribution within this period (Table 3, Fig. 6c). The first peak in this bimodal distribution was around 07:00, with a slight decrease in rates until 12:00, followed by a second peak between 14:00–16:00 (Fig. 6c). Dive rates decreased linearly after this time (i.e., into the night), and a similar increase was seen leading up to the first morning peak (Fig. 6c). Individual-level variation was significant as in the other models (Table 3), but there was more conformity to this bimodal functional relationship with dive rate and time-of-day, unlike the models for depth and duration (Fig. 6c). This includes nearly all individuals from the MHI population and one individual from the open-ocean population. Four individuals (three NWHI, one open-ocean) had higher hourly dive rates in the morning (i.e., had unimodal relationships), while one individual (NWHI population) had higher dive rates at night (Fig. 6c). There was evidence for an effect of moon phase on hourly dive rate (Table 3), although the predicted effect was minimal (Fig. 6c).

#### Near-seafloor diving behavior

**Fig. 7:**
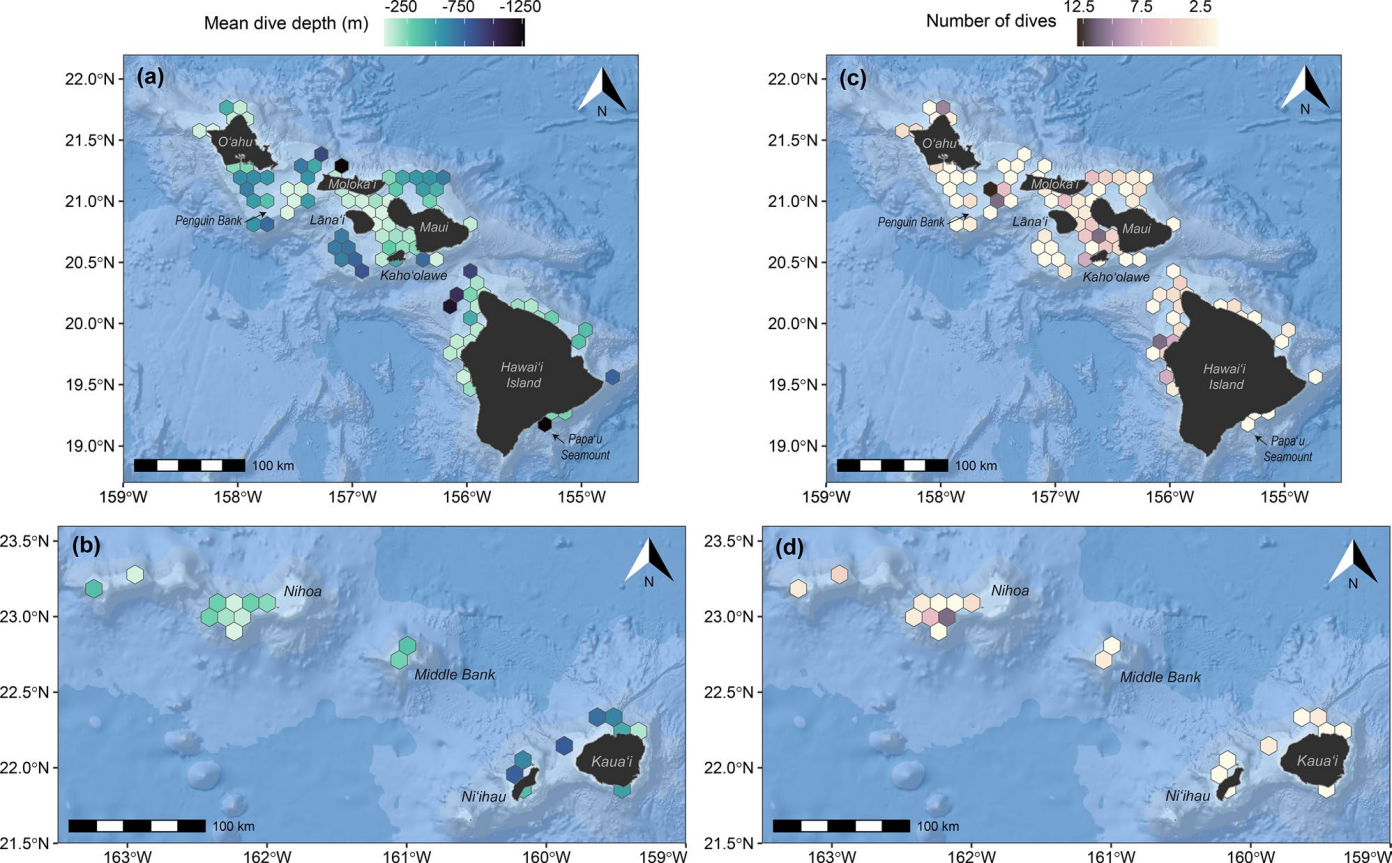
Gridded spatial distribution of probable near-seafloor dives for MHI and NWHI insular false killer whales. (**a, b**) Mean dive depth; and (**c, d**) Number of dives. Hexagon cell size (for those not intersecting with land) is 150 km^2^

All tagged individuals from the MHI and NWHI populations had dives that initially appeared to be close to the seafloor, but for many dives, this appeared to be an artifact of tag location error (i.e., high variation in seafloor depth across 20 imputed dive positions; Figure S7, S8, Additional File 2). Out of the 1620 candidate dives (i.e., those with positional uncertainty < 4 km) from all tagged insular whales, there were 287 dives that were considered probable near-seafloor dives (i.e., standard deviation of seafloor depths < 100 m, max depth < 100 m from seafloor). The proportion of probable near-seafloor dives out of all candidate dives was low (mean = 16.9%, Table 4). Five individuals had proportions greater than this mean, ranging from 21% to 48% of all candidate dives as probable near-seafloor dives (Table 4). The overall mean dive depth and duration for dives that were likely at or close to the seafloor were 321 m and 7.4 min, respectively, and ranged from shallow dives (52 m) to very deep dives (1272 m), with all but three of the thirteen insular individuals having probable seafloor dives deeper than 500 m (Table 4). Most seafloor dives were square- or u-shaped, but this varied among individuals (Table 4). Spatially, seafloor dives occurred on shallow banks, shelves, and both gradual and steep slopes (Fig. 7). The most seafloor dives occurred on Penguin Bank, along the north and west shelf/slopes of Oʻahu, and in the leeward waters of Maui Nui, all of which are relatively shallow areas ( < 500 m; Fig. 7). Three individuals, all from the MHI population, had probable near-seafloor dives exceeding 1000 m: PcTag026 had one 1272 m dive recorded along the steep slope off southeastern Hawaiʻi Island, adjacent to the Papaʻu Seamount (Fig. 7); PcTag032 dove to 1168 m around the north Molokaʻi submarine canyons; and both PcTag032 and PcTag099 dove to 1264 m and 1020 m, respectively, along the deep slope off northwest Hawaiʻi Island (Fig. 7). Temporally, median probable seafloor dive rates were highest during the day and at night, while the maximum rates occurred during dawn; however, there was high variability in dive rates by time-of-day, as several individuals had near-seafloor dives during some diel categories but not others (Table 4; Figure S9, Additional File 2). Median depths of near-seafloor dives were slightly deeper at dawn and dusk than night or day, although the distribution of dive depths and durations were broadly similar across diel categories (Table 4; Figure S9, Additional File 2).

**Table 4:** Summary of dives (≥50 m, ≥ 2 min) estimated to be close to the seafloor for insular false killer whales

Population	Tag ID	# candidate dives (% total)		Probable near-seafloor dives					
			# dives (% candidate)		Median (range) dive depth (m)	Median (range) dive duration (min)	Median (min*) ratio dive depth to seafloor depth	# Square/U/V- shaped dives	# dawn/day/dusk/night dives
*MHI*	PcTag026	53 (76.8)	5 (9.4)		584 (536–1272)	10.9 (7.9–14.7)	0.99 (0.87)	0/5/0	0/3/1/1
	PcTag028	204 (72.6)	43 (21.1)		64 (55–520)	6.0 (2.1–11.2)	0.89 (0.46)	38/4/1	6/22/0/15
	PcTag030	267 (88.1)	23 (8.6)		248 (78–560)	6.4 (3.3–9.1)	0.90 (0.59)	10/13/0	3/9/0/11
	PcTag032	131 (77.1)	15 (11.5)		456 (116–1264)	9.6 (3.6–18.1)	0.96 (0.79)	4/10/0	0/7/2/6
	PcTag055	54 (94.7)	26 (48.1)		70 (52–752)	5.7 (3.2–13.2)	0.97 (0.61)	22/3/1	1/15/0/10
	PcTag074	84 (100.0)	5 (6.0)		432 (152–816)	8.0 (4.5–11.8)	0.93 (0.92)	4/1/0	0/4/0/1
	PcTag095	254 (96.2)	84 (33.1)		124 (52–312)	4.3 (1.9–8.6)	0.83 (0.46)	59/25/0	0/61/5/18
	PcTag099	188 (98.4)	40 (21.3)		340 (64–1012)	7.6 (2.6–14.0)	0.94 (0.58)	8/28/4	4/21/5/10
*NWHI*	PcTag035	33 (62.3)	1 (6.1)		400 (NA)	6.5 (NA)	1.00 (NA)	0/1/0	0/1/0/0
	PcTag037	86 (89.6)	3 (3.5)		768 (448–816)	11.1 (6.1–12.5)		0/3/0	0/3/0/0
	PcTag049	121 (57.1)	10 (9.1)		496 (136–768)	9.0 (5.3–12.9)	1.00 (0.60)	6/4/0	2/2/2/4
	PcTag096	68 (97.1)	22 (32.4)		109 (58–320)	5.2 (2.3–8.9)	0.90 (0.53)	21/1/0	0/17/0/5
	PcTag097	77 (97.5)	10 (13.0)		89 (60–656)	5.6 (2.6–11.9)	0.92 (0.48)	10/0/0	2/6/0/2

The number of candidate dives represents the number of dives after excluding those with estimated positional errors 4 km or greater. We considered “probable near seafloor dives” as those with a standard deviation of seafloor depths (across the 20 imputed locations) of 100 m or less, and a maximum depth difference (maximum seafloor depth minus dive depth) of 100 m or less. The ratio of median dive depth to seafloor depth represents the proportion of the water column reached by dives (values closer to 1 indicate dives approaching the seafloor)

## Discussion

Using two types of bio-logging tags, we described the vertical movements of false killer whales at both fine- and coarse-scales and in relation to ecological contexts. We found that false killer whales spend most of their time in the epipelagic zone (i.e., surface to approximately 200 m depth) and exhibit a diversity of dive types across different habitats, with substantial variation over time and across individuals and demographic groups. These findings, combined with observed spatial patterns, suggest that false killer whales adapt their vertical movements to different prey types and environmental conditions. This study fills critical species-specific knowledge gaps and reveals surprisingly deep diving behavior in false killer whales. In the following sections, we discuss the potential ecological and demographic drivers of observed inter-individual variation in vertical movements, and hypothesize how they collectively provide emerging insight into this species’ foraging ecology.

### Vertical movements: diving behavior of false killer whales

Our study presents the first comprehensive description of diving behavior for false killer whales, providing a foundation for the vertical dimension of their movement ecology. All tagged false killer whales—both short-term, fine-scale TDR and long-term, coarse-scale SPLASH-tagged—spent a high proportion of time in the top 50 m of the water column and their median dives depths were typically in the top 300 m. This preference for the upper portions of the water column aligns with visual observations of behavior, including the variety of surface-oriented prey they have been documented feeding on [36, 50], and with insights from suction-cup tagged false killer whales elsewhere [51]. Despite their clear near-surface tendencies, the SPLASH-tagged false killer whales were capable of diving much deeper and did so more often than previously assumed. All these individuals performed dives exceeding 700 m, nearly half of them performed dives deeper than 1000 m, and the maximum depth observed (1424 m) was more than twice that known for the species [51]. Such extensive vertical movements suggest false killer whales actively forage at depth. While we cannot infer definitive feeding events from SPLASH tags, similar deep diving behavior is known to occur in foraging contexts of related, similar-sized and sympatric species, such as short-finned pilot whales (*Globicephala macrorhynchus*; [94]), goose-beaked whales (*Ziphius cavirostris*, [95]), and Blainville’s beaked whales (*Mesoplodon densirostris*, [95]). Elucidating the function of these deep dives and their frequency across individual traits will require additional tag deployments, including satellite tags and other bio-loggers (e.g., those equipped with 3D accelerometers, acoustic sensors).

### Demographic contexts of movement: from individuals to populations

Individual traits such as morphology (e.g., body size), age, and sex can shape the physiological capacities for diving in marine mammals [96]. Consequently, individual traits modulate access to some prey communities at depth, thus contributing to inter-individual variation in foraging behavior (e.g., [97, 98]). We found a positive but weak correlation between relative body size (i.e., dorsal fin base length) and diving (i.e., depth and duration), and most individuals that undertook the deepest dives also had longer fin base lengths. The weak correlation is unsurprising, as nearly all tagged whales were adults and thus differences in relative body size are smaller than expected between younger individuals and adults. However, the estimated difference in body length between the smallest and largest adults could exceed one meter [63], and thus even a weak correlation in the data could indicate meaningful differences in dive capacity with body size. Additionally, while fin base length is a proxy for body size, it may not scale linearly or identically for both sexes. Males dove deeper and longer than females and those of unknown sex, but there are some caveats in these sex-based differences in diving capacities—largely driven by an outlier individual (PcTagP09) and only three of the satellite-tagged whales were known to be females and six were of unknown sex. Similar limitations preclude robust inference on age-based differences. Among all satellite-tagged individuals, only two were non-adults (juvenile and sub-adult) because adults are targeted for this type of tag due to their larger body size (and thus target area) and more predictable surfacing behavior. Ontogenetic shifts in diving behavior are thus not currently discernable but could be evaluated in the future with additional deployments on younger individuals with these tags or less invasive tag types. Further assessment of morphology-driven variation in diving behavior will also benefit from future drone-based photogrammetry (e.g., [97]).

Inter-individual variation in behavior, such as vertical movements, can also scale up to population-level foraging strategies, even among sympatric populations (e.g., [99]). Our available tag data suggest high within-population variability in vertical movements, even though our current sample size precludes a robust assessment of specializations or strategies at the population level. For example, PcTag049 primarily dove at night, a notably different diel pattern than the other four tagged NWHI false killer whales. Similarly, PcTagP09 dove deeper and much more frequently than all other tagged individuals combined, including the others from the open-ocean population. There was high variability in diving behavior within the MHI population as well, including the spatial distribution of dives, diel trends, and dive metrics. Such variation in diving behavior currently precludes generalization of population-level patterns, which will require more tag deployments. Additional genetic analyses of biopsy samples would help unravel within-population structuring (particularly for open-ocean false killer whales), and the extent to which differences in dive behavior reflect the degree of relatedness. Further, stable isotope analysis of biopsy samples from all three populations would help identify whether all populations exhibit similarities in diet breadth, or if some exhibit narrower trophic niches that reflect resource specialization (e.g., [14]).

Group-specific movement strategies can emerge from inter-individual variation in behavior [100], particularly if individuals cooperatively hunt and behaviors can be socially learned (e.g., [101]). Insights on social cluster contexts emerge from the available tag data, even though identifying cluster-level strategies in MHI false killer whales would also necessitate additional tag deployments. Most SPLASH-tagged MHI false killer whales were members of Cluster 3, and we found moderate variability in dive metrics within this social cluster, including diel trends. The number of dives was the most spatially constrained metric, and regions with the most dives overlapped with known high-use areas for Cluster 3 [49, 102]. However, individuals from other clusters also spent time and dove > 50 m in these habitats, and Cluster 3 high-use areas have high overlap with that estimated for the overall population [71]. We additionally highlight the frequent near-seafloor diving behavior of PcTag055 (nearly 50% of all dives) as a potential indicator of cluster-level foraging tactics. This whale is the only individual in our dataset that belongs to Cluster 4 of the MHI population. Compared to the other social clusters, Cluster 4 has the most restricted space use, primarily using habitats within Maui Nui [41, 49, 102]. In contrast, high-use areas for the other clusters are found along the windward sides of Maui Nui, Oʻahu, and off northern Hawaiʻi Island, and individuals from two of these clusters are known to move widely throughout the main Hawaiian Islands [40, 102]. Variation in persistent organic pollutant levels and stable isotopes also suggest that foraging habitats may slightly differ among the social clusters [43]. Foraging site fidelity is often correlated with foraging specializations when prey availability is consistent and abundant (e.g., [103]), and perhaps this is the case for Cluster 4. Indeed, the high-use area of this social cluster also contains a large proportion of bottom fish (e.g., green jobfish or uku, *Aprion virescens*) habitat in the MHI region [104]; this shallow water habitat could additionally make near-seafloor dives more accessible. However, tagged whales from other clusters also exhibited near-seafloor diving behavior, and the deployment duration for PcTag055 was relatively short (approximately 1 week). Additional SPLASH tag deployments coupled with more comprehensive diet information will help discern social cluster-level foraging behavior.

### Ecological contexts of movement: prey and niche partitioning

Across all tagged individuals combined, we found that false killer whales predominantly occupy the epipelagic zone. While definitive prey captures cannot be inferred from either TDR or satellite tags, visual observations of foraging on surface-oriented epipelagic fish support these findings [36, 105]. We observed one of the whales chasing a mahimahi shortly after being equipped with a TDR tag, and its use of the near-surface waters ( < 50 m) during this time validates foraging in the epipelagic zone from tag-derived measurements. Mahimahi spend most of their time in the top 50–150 m of the water column but will use much shallower waters when associated with floating objects [106], which false killer whales have regularly been observed hunting mahimahi around (RWB, personal observation). The lower ascent rates (compared to descent rates) reported by the TDR tags could reflect a hunting tactic for such surface-oriented prey—or a predator avoidance tactic as suggested for beaked whales (e.g., [107])—where false killer whales slow their ascent rates to visually scan for prey (or predators) near the surface. This pattern could also be a mechanism for preventing the formation of gas bubbles in blood or tissues [108], however, this hypothesis is more relevant for deeper dives (e.g., [109, 110]). Combined with the high proportion of time spent above 50 m, the significant relationship between dive depth and mixed layer depth in the multivariate model additionally suggests foraging on prey that occupy the epipelagic zone, or prey that respond to variation in the mixed layer (although this only explained 1% of the model deviance). Many mid- and upper-trophic level epipelagic fish included in false killer whales’ diet are known to spend high proportions of their time in the surface mixed layer and epipelagic zone more broadly (e.g., ʻahi, [75, 82]). Other large game fish, such as bigeye tuna or ʻahi poʻonui (*Thunnus obesus*), occupy deeper depths (300–500 m) during the day, with excursions to the surface mixed layer within this time period [76]. Thus, together with the temporal findings (discussed further in subsequent sections), false killer whale dives within the 200–300 m depth range could be targeting prey moving between epi-/mesopelagic zones such as ʻahi poʻonui, or pursuing prey within the epipelagic zone that attempt to flee by diving deeper (e.g., ʻahi, [75]). Overall, these findings indicate that information inferred from surface observations of false killer whale predation events or prey sharing likely reflect the majority of their diet, although given their observed deep diving behavior, surface observations are not reflective of their entire diet.

Tagged false killer whales exhibited a diversity of dive types when they were not using near-surface waters or diving within the epipelagic zone. Deeper, albeit comparatively infrequent dives outside of the epipelagic zone could reflect foraging on other prey species or types. For example, false killer whales have been observed feeding on monchong (lustrous pomfret or mukau, *Eumegistis illustris*; [36]) which occur along deep slopes ( > 900 m) and near seamounts [111], and moonfish or opah (*Lampris guttatus*; [50]) which utilizes the mesopelagic zone [77]. The island-associated mesopelagic boundary layer occupies the 400–700 m depth zone during the day [112] and it is possible that dives to these depths (or deeper) could reflect foraging for such mesopelagic or interzonal species knowing that false killer whales have consumed squid that occupy these vertical habitats (from stomach contents: diamondback squid, *Thysanoteuthis rhombus,* and purpleback flying squid, *Sthenotheuthis oualaniensis*; K. West personal communication; [113, 114]. Although inconclusive, probable predation events on tagged yellowfin tuna in Hawaiian waters—inferred from isolated, uncharacteristically deep dives of up to 1500 m [75]—raise the possibility that they were fleeing from predators that can pursue prey at depth, including false killer whales.

We documented probable seafloor dives in all MHI and NWHI false killer whales, and the depth of near-seafloor dives were variable. Penguin Bank, where these dives were most common and limited to within 200 m, is known critical habitat for bottom fish, such as uku [115], which MHI false killer whales have been observed feeding on [50]. Probable near-seafloor dives also occurred in deeper habitats, including steep slopes, gradual deep slopes, and submarine canyons, reflecting potential pursuit of deep slope associated prey (e.g., monchong; [111]). Submarine canyons in Hawaiʻi are biological hotspots, where dynamic interactions between currents and topography enhance local nutrient availability and thus aggregate a variety of mobile fish [116]. The submarine canyons off Oʻahu and Moloka‘i in particular are known to have increased species richness relative to nearby slopes [116]. Thus, the probable seafloor dives near these features could potentially reflect foraging on these localized prey aggregations. This near-seafloor diving tactic does not appear to comprise a high proportion of their diving activity (with one exception discussed above), although it is possible that it occurs more frequently than reported here due to the restrictions applied for location uncertainty. Other bio-loggers, such as those equipped with acoustic sensors or cameras, could inform the function of these near-seafloor dives.

Our multivariate findings suggest that temporal cycles, serving as proxies of prey movement, are not a primary driver of false killer whale diving behavior. Although we found evidence for a common trend of diel and lunar patterns in dive depth where dives were deeper (by approximately 50 m) in the middle of the day and during full moons, the shape of this diel trend varied significantly among individuals. Several individuals had contrasting patterns in dive depth and duration with time-of-day (e.g., nocturnal versus diurnal, some deeper during dawn, others during dusk), even within a population. Minamikawa et al. [51] did report deep dives ( > 200 m) predominantly occurring during the daytime, although their sample of dive data was only for one individual for three days. Diel patterns in hourly dive rate, however, were more generalizable across all tagged false killer whales, where dives > 50 m occurred most frequently during daytime (except for one NWHI individual) with peaks just after dawn and prior to dusk. Increased frequency of dives during the day, but across variable depths and durations, could suggest that false killer whales forage deeper more often during the daytime but on a variety of prey that may or may not exhibit diel vertical migrations. High variation and complex relationships in diel patterns have been observed in other marine predators that exploit a diversity of prey types and whose availability may change across habitats (e.g., belugas, *Delphinapterus leucas,* [117] leatherback sea turtles, *Dermochelys coriacea,* [29]). Further, a recent study documented the occurrence of a deep, non-migratory micronekton (fish, squid) layer in the leeward waters of Hawaiʻi Island [118] false killer whales may exploit this non-migratory prey field, which could additionally explain the high variability in their diving behavior.

Niche partitioning among sympatric predators may also influence diel patterns in false killer whale diving behavior. Twenty-six species of cetacean have been documented around the Hawaiian Archipelago, eleven of which are odontocetes that are resident year-round and geographically overlap with false killer whales [71, 105]. Several sympatric predators, including spinner dolphins, rough-toothed dolphins, short-finned pilot whales, pantropical spotted dolphins, and melon-headed whales, exhibit vertical movements that appear to track diel vertically migrating prey [25, 56, 94, 119, 120], which contrasts to the high variation and weak diel patterns we found for false killer whales. Some of these studies have also found strong effects of lunar phase on diving behavior [25, 94], which again differs from our findings. Thus, it is possible that these sympatric delphinids fill a niche as predators on the DSL prey community that false killer whales only opportunistically exploit. This could be additionally inferred by contrasting movement and space use patterns observed in these other species: the high movement rates and frequency of inter-island movements exhibited by false killer whales [40, 121] are either infrequent or rare in the other resident odontocetes, which typically exhibit site fidelity to only one or few islands [71, 105]. Prey species comprising the diel vertically migrating DSL are typically less mobile than large epipelagic prey, and thus the stronger site fidelity and diel foraging behavior of sympatric predators could point to spatial and temporal partitioning with more generalist and higher trophic level false killer whales.

Our multivariate model results shed further light on the importance of dynamic oceanographic variables on false killer whale vertical movement behavior. On average, false killer whales dove deeper and longer when lagged surface chlorophyll-a concentrations were low and when surface current magnitude was high. Unlike the dive depth model results, the estimated trend between dive duration and lagged chlorophyll-a concentrations plateaued at higher values. These findings suggest that when surface productivity is low, false killer whales dive deeper (and thus longer), presumably targeting alternative prey deeper in the water column. High surface chlorophyll-a concentrations could also indicate reduced visibility in near-surface waters, which could subsequently affect prey pursuit and capture efficiency. Variance in chlorophyll-a concentrations could be confounded by seasonal climatic variation; however, the Central North Pacific lacks strong seasonal variability in primary productivity [122], making prey dynamics or visibility more plausible explanations for our findings. Areas with stronger horizontal current magnitude can indicate the presence of oceanographic fronts or edges of mesoscale eddies, which are typified by vertical mixing and known to be hotspots of biological activity [123, 124]. Other pelagic predators have been observed modifying their vertical movements and foraging at these features (e.g., seabirds, [125] pinnipeds, [126]), and thus it is plausible that our results on false killer whales reflect similar behavior. Collectively, the diversity of prey and dive types, our findings on epipelagic tendencies and variable diel and lunar patterns in diving behavior, and the observed relationships between vertical movements and oceanographic variables all support a hypothesis for an ecologically driven, flexible foraging strategy.

## Conclusions

Our study provides the first comprehensive description of false killer whale diving behavior and enhances understanding of their movement ecology. We highlight that our study takes place in a unique marine ecosystem—the Hawaiian Archipelago—where long-term multi-species studies have developed critical ecological context for interpreting false killer whale vertical movement behavior. Both fine-scale, short duration and coarse-scale, intermediate-long duration tags showed that false killer whales primarily occupy the epipelagic zone, a vertical habitat that many large, upper trophic level prey inhabit. Satellite tags additionally documented dives to a wide range of depths—including surprisingly deep ( > 1000 m) diving behavior and a record maximum of 1424 m—and within a variety of habitats. Temporal proxies of prey availability do not appear to shape false killer whale diving behavior; inter-individual variation in diel trends and proxies of biological productivity (i.e., surface chlorophyll-a concentration, current magnitude) and upper trophic level fish habitat (i.e., mixed layer depth) captured the most variation in the data, albeit with small effect sizes. There was also high variability in dive metrics among individuals of different age/sex classes, social clusters, and populations. These findings, paired with false killer whales’ diverse diet and sympatry with predators known to exploit the DSL, support the hypothesis of a flexible, ecologically driven foraging strategy in this social pelagic predator. While additional tag deployments are needed to better characterize drivers of behavioral variability and more explicitly link diving with foraging behavior, our results provide emerging insights into the role of ecological context on the vertical movements of false killer whales. Our findings also serve as a foundation for interpreting false killer whale movements in the context of various conservation-oriented goals (e.g., energetics, important foraging habitats, fisheries interactions), particularly for the endangered MHI population that has declined over the recent decade [49].

## Supplementary Information

Below is the link to the electronic supplementary material.

Supplementary Material 1

Supplementary Material 2

## Data Availability

Tag data used in this study can be visualized on Movebank (https://movebank.org) and are available from the corresponding author on reasonable request. All code developed to process, analyze, and visualize the data in this study are available at https://github.com/makratofil/fkw-dive-behavior.

## Abbreviations

DSL: Deep scattering layer
MHI: Main Hawaiian Islands
NWHI: Northwestern Hawaiian Islands
CRC: Cascadia Research Collective
PIFSC: Pacific Islands Fisheries Science Center
TDR: Time-depth recorder
LIMPET: Low-Impact Minimally-Percutaneous External-electronics Transmitter
CTCRW: Continuous-time correlated random walk
GAMM: Generalized additive mixed effects model
AIC: Akaike’s Information Criterion
CV: Coefficient of variation

## Footnotes

1 https://www.gebco.net/data_and_products/gridded_bathymetry_data/gebco_30_second_grid/

2 https://data.marine.copernicus.eu/product/OCEANCOLOUR_GLO_BGC_L4_MY_009_104/description

3 https://data.marine.copernicus.eu/product/GLOBAL_MULTIYEAR_PHY_001_030/description

4 https://www.soest.hawaii.edu/hmrg/multibeam/bathymetry.php

5 https://www.soest.hawaii.edu/hmrg/multibeam/grids.php
