# Supplementary material for: Ecological contexts of diving behavior in Hawaiian false killer whales
Source: Mov Ecol. 2026 Mar 6;14:17. doi: 10.1186/s40462-026-00630-4 (PMC13003685; doi:10.1186/s40462-026-00630-4)
Supplement: Supplementary file 2 — Supplementary Material 2 [file 40462_2026_630_MOESM2_ESM.docx]

Additional File 2: Supplementary tables and figures for **“Ecological contexts of diving behavior in Hawaiian false killer whales”**

Michaela A. Kratofil^1,2*^, Jacquelyn F. Shaff^2,3^, Holly K. Hoffbauer^1,4^, Mauricio Cantor^1^, Marie C. Hill^5,6^, Robin W. Baird^2^

*^1^Marine Mammal Institute, Department of Fisheries, Wildlife, and Conservation Sciences, Oregon State University, Newport, OR, United States*

*^2^Cascadia Research Collective, Olympia, WA, United States*

*^3^Marine Mammal Commission, Bethesda, MD, United States*

*^4^University of Alaska Southeast, Juneau, AK, United States*

*^5^Cooperative Institute for Marine and Atmospheric Research, Research Corporation of the University of Hawaiʻi, Honolulu, HI, United States*

*^6^NOAA Pacific Islands Fisheries Science Center, Honolulu, HI, United States*

**Corresponding author (michaela.kratofil@oregonstate.edu)*

Table S1. Summary of primary analytical objectives and corresponding datasets used in the study.

| **Analysis** | **Tag type** | **Description of data used** |
| --- | --- | --- |
| Description of short-term, fine-scale dive behavior, comparisons across diel categories | TDR | All available dives (0- 230 m) |
| Description of long-term coarse-scale dive behavior, comparisons across demographic groups, diel categories, and spatial distributions | SPLASH | All available dives (≥ 50 m, ≥ 2 min) |
| Analysis of spatial and temporal predictors of dive behavior metrics (GAMMs) | SPLASH | All dives (≥ 50 m, ≥ 2 min) with estimated spatial uncertainty < 4 km. |
| Analysis of temporal predictors of dive behavior metrics (GAMMs; sensitivity assessment for data restriction above) | SPLASH | All available dives (≥ 50 m, ≥ 2 min) |
| Identifying probable near-seafloor dives | SPLASH | Dives (≥ 50 m, ≥ 2 min) from MHI and NWHI individuals only, with estimated spatial uncertainty < 4km; multiple imputations of dive locations restricted to dives with ± 100 m SD of seafloor depth |

Table S2. Summary of dive information from five TDR tags deployed on false killer whales from the main Hawaiian Islands. Time at <50 m dives (%) represents the time the TDR individuals would have as “time at surface” as defined for the SPLASH satellite tags. PcTDR04 had a few dives exceeding the maximum value of the tag’s depth sensor (approximately 230 m), and thus, while the dive durations are accurate, these dives were deeper than the reported value (indicated by the asterisk). Relative velocity is reported as uncalibrated units, given known influence of tag location and likely body size on swim speed measurements (see Baird et al. 2001).

|  |  |  | Metric mean (SD) / median / maximum | | | | |
| --- | --- | --- | --- | --- | --- | --- | --- |
| Tag ID | Time at <50 m dives (%) | Number of dives (≥10 m) | Dive depth (m) | Dive duration (min) | Relative velocity (uncalibrated units) | Descent rate (m/s) | Ascent rate (m/s) |
| PcTDR01 | 100.0 | 7 | 13(5) / 11 / 22 | 1.5(0.4) / 1.7 / 2.0 | 0.5(0.4) / 0.5 / 3.5 | 0.5(0.2) / 0.4 / 0.9 | 0.7(0.3) / 0.6 / 1.1 |
| PcTDR02 | 99.7 | 260 | 17(7) / 16/ 52 | 1.4(0.8) / 1.3 / 4.0 | 0.5(0.5) / 0.4 / 6.8 | 1.3(0.7) / 1.2 / 5.8 | 0.7(0.7) / 0.6 / 5.8 |
| PcTDR03 | 99.2 | 35 | 17(10) / 13 / 53 | 1.1(0.5) / 0.9 / 3.0 | 1.9(0.7) / 1.9 / 6.9 | 1.3(0.6) / 1.2 / 2.6 | 0.9(0.3) / 1.0 / 1.9 |
| PcTDR04 | 95.7 | 367 | 24(31) / 18 / 234* | 2.4(1.7) / 1.9 / 12.0 | 0.9(0.9) / 0.8 / 6.6 | 0.8(0.5) / 0.7 / 4.8 | 0.5(0.4) / 0.5 / 5.1 |
| PcTDR05 | 97.7 | 47 | 23(26) / 19 /180 | 1.8(0.7) / 1.7 / 3.6 | - | 1.0(0.6) / 0.9 / 3.1 | 0.7(0.6) / 0.6 / 3.6 |

Table S3. Summary of behavior data from SPLASH satellite tag deployments on both insular (MHI = Main Hawaiian Islands; NWHI = Northwestern Hawaiian Islands) and open-ocean false killer whales. Dive rate was calculated by dividing the total number of dives by the total duration of behavior log data (i.e., summed durations of surface and dive records, excluding gaps). All dive metric values are restricted to those dives of 50 m or deeper and 2 min or longer.

| Population | Tag ID | % Time in “surface” periods (< 50 m) | Total # dives ≥ 50 m, ≥ 2 min | Dive depth (m) mean (SD) / median / max | Dive duration (min) mean (SD) / median / max | Dive rate (# dives ≥ 50 m/h) | # dives for spatial analyses (< 4 km error) |
| --- | --- | --- | --- | --- | --- | --- | --- |
| MHI |  |  |  |  |  |  |  |
|  | PcTag026 | 95.4 | 69 | 384 (259) / 424 / 1272 | 6.8 (2.9) / 6.4 / 14.7 | 0.41 | 53 |
|  | PcTag028 | 92.4 | 281 | 181 (150) / 112 / 728 | 6.1 (2.2) / 5.8 / 12.5 | 0.75 | 204 |
|  | PcTag030 | 94.6 | 303 | 251 (156) / 204 / 992 | 5.1 (1.9) / 4.9 / 11.2 | 0.63 | 267 |
|  | PcTag032 | 95.8 | 170 | 362 (269) / 316 / 1264 | 7.1 (3.6) / 6.6 / 18.7 | 0.35 | 131 |
|  | PcTag055 | 96.3 | 57 | 224 (223) / 128 / 816 | 6.0 (2.8) / 5.1 / 13.2 | 0.36 | 54 |
|  | PcTag074 | 97.1 | 84 | 221 (187) / 152 / 864 | 4.5 (2.4) / 3.8 / 11.8 | 0.40 | 84 |
|  | PcTag095 | 97.1 | 264 | 143 (96) / 120 / 752 | 4.2 (1.5) / 4.0 / 8.6 | 0.42 | 254 |
|  | PcTag099 | 97.0 | 191 | 277 (234) / 208 / 1136 | 5.8 (2.7) / 5.4 / 14.0 | 0.31 | 188 |
| NWHI |  |  |  |  |  |  |  |
|  | PcTag035 | 96.5 | 53 | 369 (207) / 376 / 1040 | 6.2 (3.2) / 5.9 / 16.7 | 0.34 | 33 |
|  | PcTag037 | 96.2 | 96 | 298 (225) / 260 / 928 | 5.8 (3.2) / 4.8 / 15.2 | 0.39 | 86 |
|  | PcTag049 | 93.1 | 212 | 253 (186) / 172 / 1104 | 6.9 (2.8) / 6.4 / 18.2 | 0.61 | 121 |
|  | PcTag096 | 93.4 | 70 | 189 (146) / 132 / 640 | 5.2 (2.0) / 5.1 / 12.7 | 0.76 | 68 |
|  | PcTag097 | 95.5 | 79 | 252 (200) / 164 / 832 | 5.8 (2.8) / 5.3 / 13.6 | 0.46 | 77 |
| Open-ocean |  |  |  |  |  |  |  |
|  | PcTag090 | 99.0 | 45 | 219 (196) / 132 / 896 | 5.0 (2.2) / 4.4 / 11.2 | 0.12 | 42 |
|  | PcTag092 | 96.4 | 32 | 295 (170) / 296 / 688 | 6.2 (2.5) / 6.2 / 12.4 | 0.36 | 30 |
|  | PcTagP09 | 89.5 | 106 | 780 (450) / 744 / 1424 | 11.9 (5.0) / 13.4 / 19.1 | 0.53 | 102 |

Table S4. Comparison of generalized additive mixed effects model performance between two different random effect structures (individual tag ID; random intercept versus random smooth) for each modeled dive behavior metric. Percent deviance explained and conditional AIC values are bolded for the highest performing model structure for each dive metric. Full model sets include both temporal and spatial predictors.

| Model set | Modeled dive behavior metric | Random effect structure | % deviance explained | AIC |
| --- | --- | --- | --- | --- |
| Temporal predictors only | Dive depth | Random intercept | 27.2 | 26962.6 |
|  | Dive depth | Random smooth | **30.3** | **26916.4** |
|  | Dive duration | Random intercept | 24.2 | 26868.9 |
|  | Dive duration | Random smooth | **26.2** | **26851.8** |
|  | Hourly dive rate | Random intercept | 10.1 | 7788.4 |
|  | Hourly dive rate | Random smooth | **14.7** | **7672.6** |
|  |  |  |  |  |
| Full (spatial & temporal predictors) | Dive depth | Random intercept | 30.2 | 22646.5 |
|  | Dive depth | Random smooth | **32.2** | **22633.9** |
|  | Dive duration | Random intercept | 26.3 | 22570.3 |
|  | Dive duration | Random smooth | **28.0** | **22567.4** |

Table S5. Outputs of generalized additive mixed effects models examining relationships between dive behavior metrics and temporal predictors only. All dives (n =2,112) were included in these models.

| Model | Covariate | EDF | F-value | p-value | Deviance explained (%) by covariate |
| --- | --- | --- | --- | --- | --- |
| Dive depth | Time-of-day (HST) | 2.410 | 4.339 | <0.001 | 0.02 |
|  | Moon phase | 2.033 | 4.044 | 0.001 | 0.06 |
|  | Random: time-of-day x ID | 40.124 | 4.454 | <0.001 | 30.21 |
|  | Total | - | - | - | 30.30 |
|  | | | | | |
| Dive duration | Time-of-day (HST) | 0.938 | 0.516 | 0.017 | 0.01 |
|  | Moon phase | 0.014 | 0.001 | 0.799 | 0.04 |
|  | Random: time-of-day x ID | 33.649 | 4.558 | <0.001 | 26.14 |
|  | Total | - | - | - | 26.20 |

Table S6. Outputs of generalized additive mixed effects models examining relationships between dive behavior metrics and temporal and spatial predictors, but with data from PcTagP09 excluded (n = 1,673 dives). For dive depth and duration models, dives with positional uncertainty of 4 km and greater were removed prior to analyses. All dives were used for the hourly dive rate model (n = 2,006 dives). The percent deviance explained by covariates for the hourly dive rate model are not included, as there is currently no functionality to obtain this metric from a model that includes an offset term. EDF = estimated degrees of freedom.

| Model | Covariate | EDF | F-value | p-value | Deviance explained (%) by covariate |
| --- | --- | --- | --- | --- | --- |
| Dive depth | Time-of-day (HST) | 2.403 | 6.164 | <0.001 | 0.00 |
|  | Moon phase | 1.760 | 1.718 | 0.035 | 0.07 |
|  | Slope | 1.980 | 1.918 | 0.012 | 0.16 |
|  | Current magnitude | 2.040 | 5.500 | <0.001 | 1.10 |
|  | Chlorophyll-a (30-d lag) | 3.414 | 4.160 | 0.001 | 1.01 |
|  | Mixed layer depth | 2.044 | 2.842 | <0.001 | 0.27 |
|  | Random: time-of-day x ID | 28.321 | 1.767 | <0.001 | 18.49 |
|  | Total | - | - | - | 21.10 |
|  | | | | | |
| Dive duration | Time-of-day (HST) | 0.583 | 0.244 | 0.084 | 0.07 |
|  | Moon phase | 0.006 | 0.001 | 0.508 | 0.01 |
|  | Slope | 0.004 | 0.000 | 0.786 | 0.01 |
|  | Current magnitude | 0.839 | 1.246 | 0.013 | 0.33 |
|  | Chlorophyll-a (30-d lag) | 3.072 | 2.342 | 0.016 | 0.18 |
|  | Mixed layer depth | 0.008 | 0.001 | 0.452 | 0.18 |
|  | Random: time-of-day x ID | 26.011 | 1.826 | <0.001 | 14.86 |
|  | Total | - | - | - | 15.50 |
|  |  |  |  |  |  |
| Hourly dive rate | Time-of-day (HST) | 2.912 | - | <0.001 | - |
|  | Moon phase | 2.171 | - | 0.006 | - |
|  | Random: hour-of-day x ID | 41.73 | - | <0.001 | - |
|  | Total | - | - | - | 15.0 |


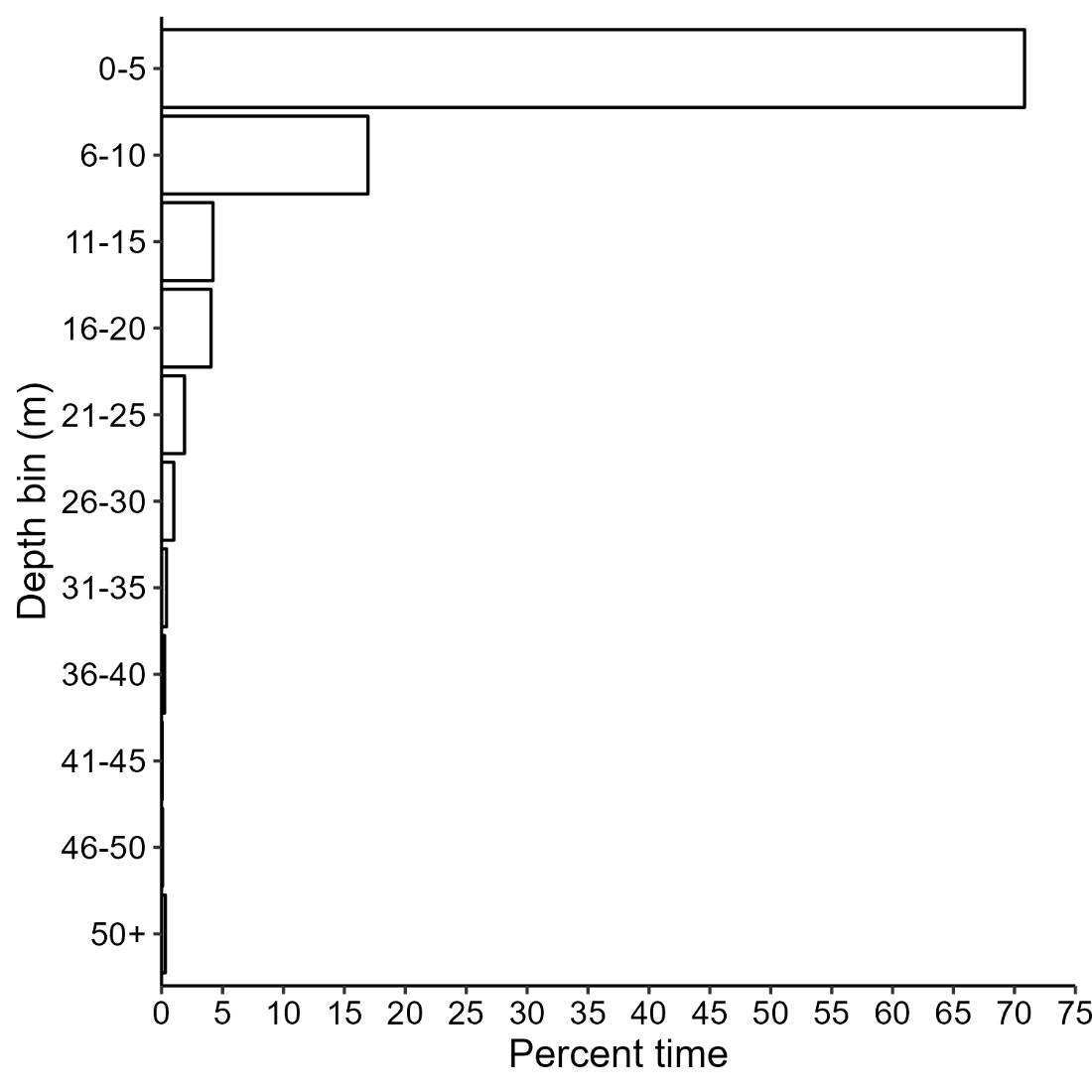


Figure S1. Proportion of total summed time spent at depth from five TDR tagged false killer whales belonging to the Main Hawaiian Islands population. Note: the last depth bin represents dives 50 m or deeper.


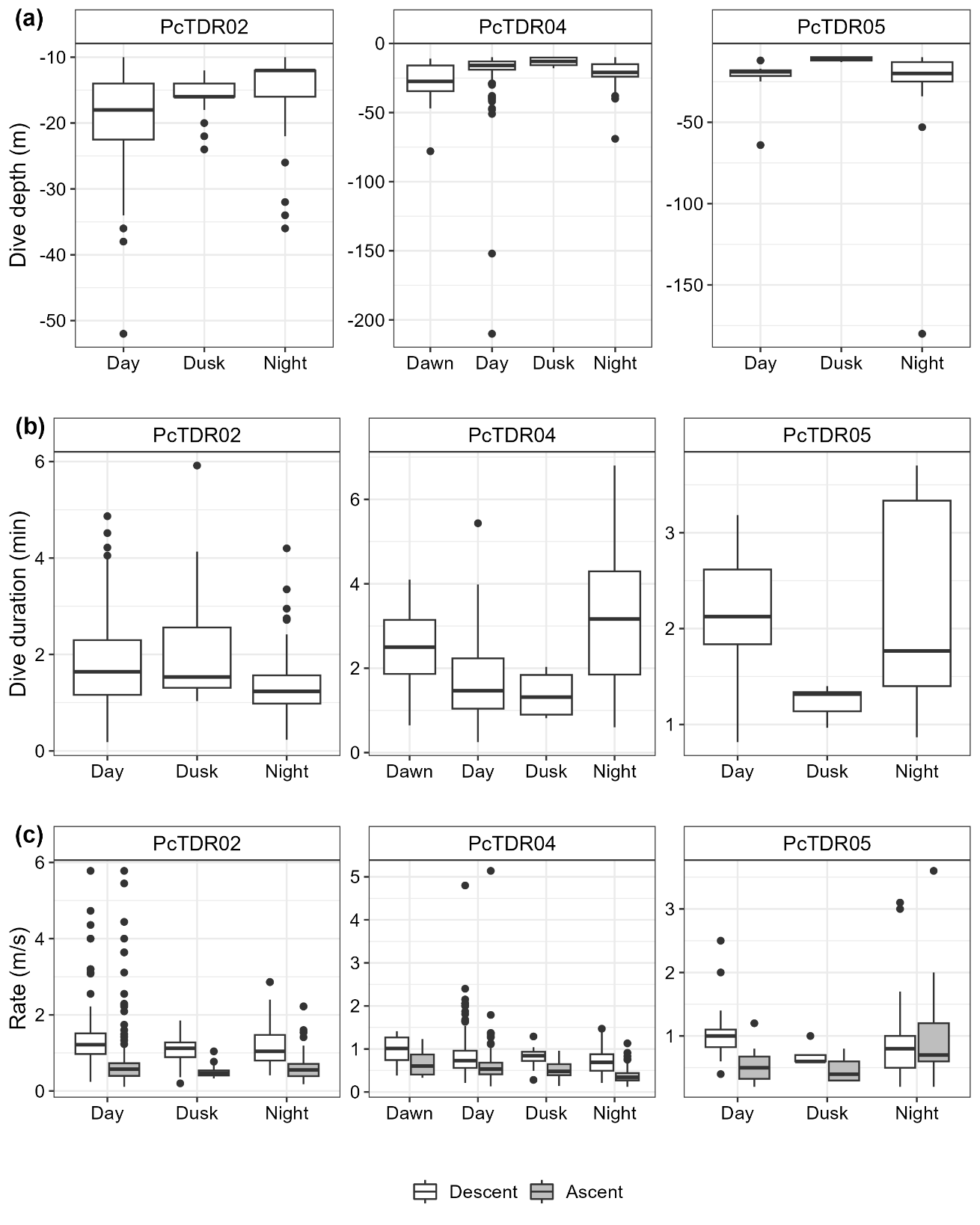


Figure S2. Box plots of (a) dive depth, (b) duration, and (c) ascent and descent rates across time of day (for dives ≥ 10 m) for PcTDR02, PcTDR04, and PcTDR05. Middle line shows median value, lower and upper lines of the boxes show 25^th^ and 75^th^ percentiles, respectively. The ends of the vertical line indicate minimum and maximum values, and outliers are indicated by black points.


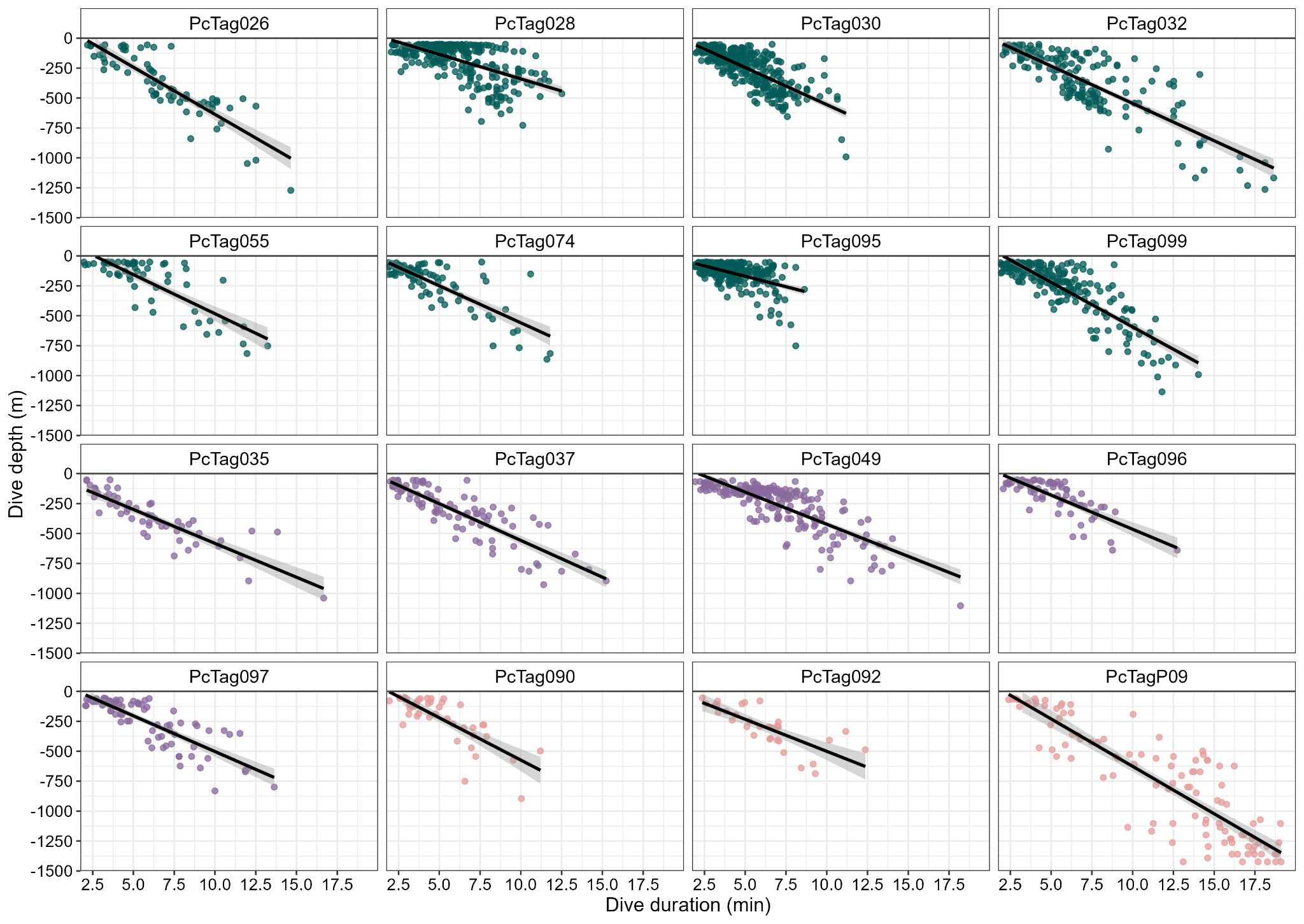


Figure S3. All dives (50 m or deeper, 2 min or longer) from SPLASH-tagged false killer whales by dive depth (y-axis, meters) and dive duration (x-axis, minutes). Panels are separated by individual, and points are colored by population (green = main Hawaiian Islands; purple = Northwestern Hawaiian Islands; pink = open-ocean). A linear smooth line (estimated from *ggplot2*’s geom_smooth() function) is shown for each individual.


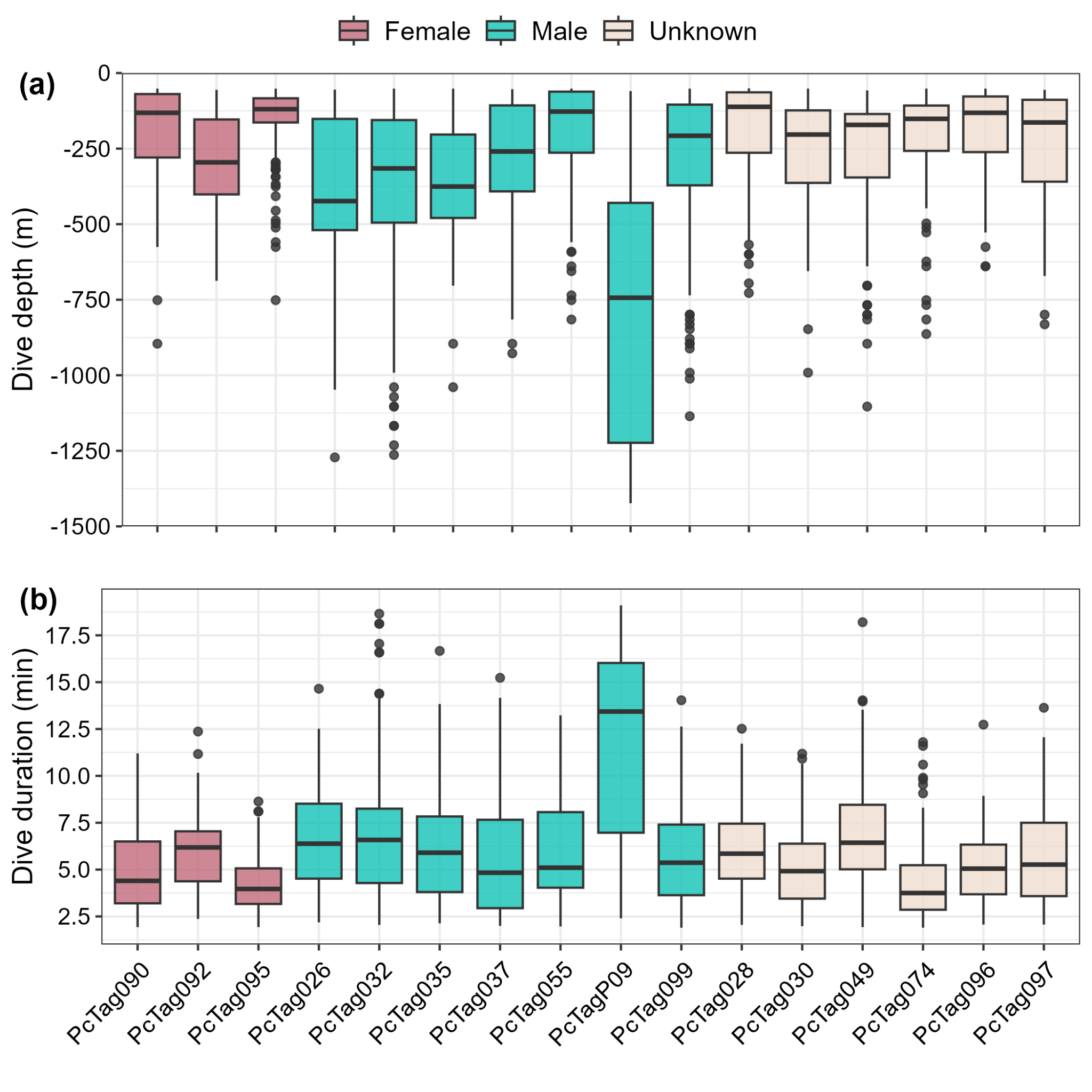


Figure S4. Boxplots of (a) dive depth and (b) duration of SPLASH-tagged false killer whales, colored by sex. The middle line in each box represents the median, the lower and upper extents of the box the first and third quartiles (respectively), the lower and upper whiskers the minimum and maximum (quartiles +/- 1.5*interquartile range), and any values outside of the box/whiskers are shown as points. See Table 1 for information on population identity.


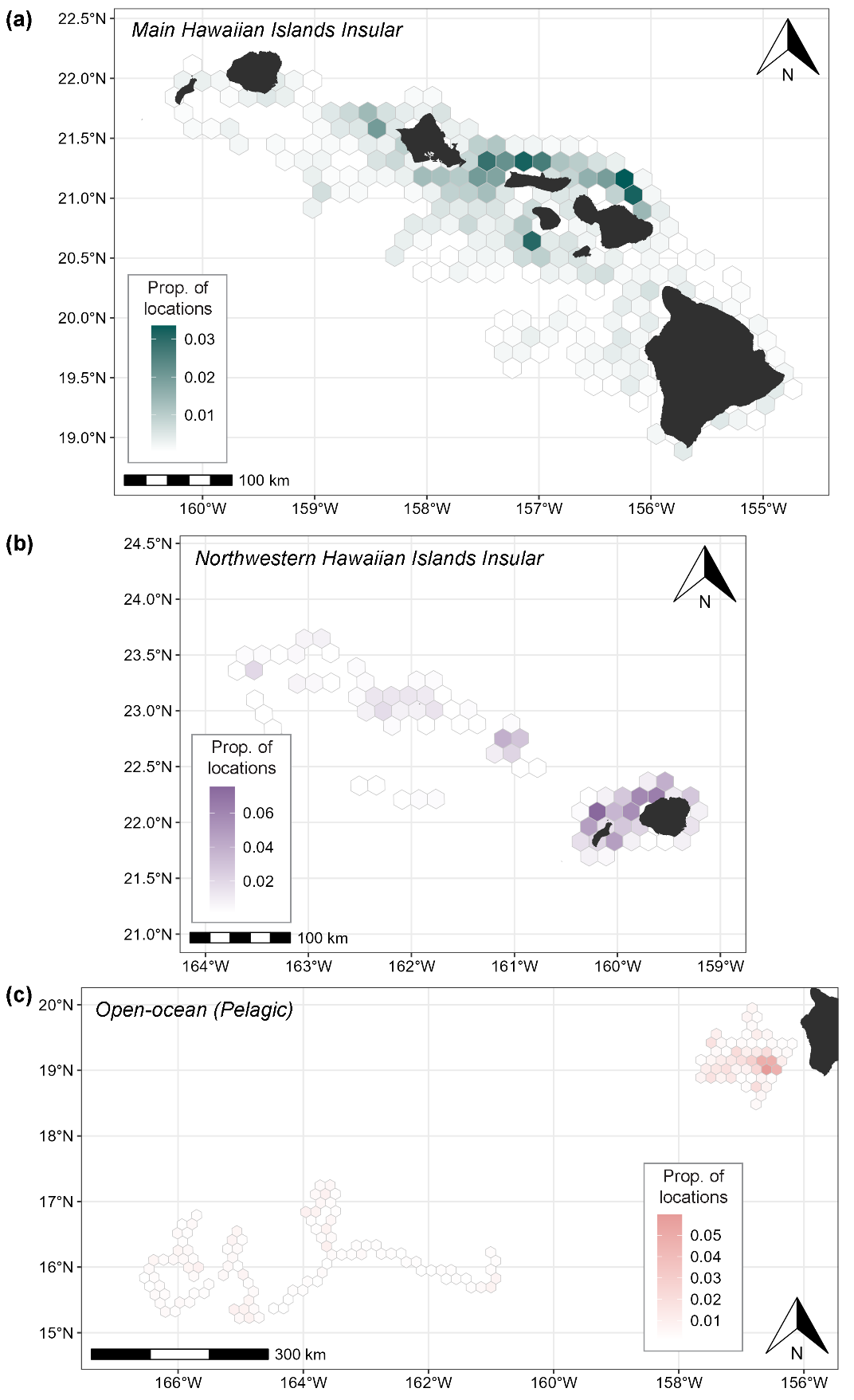


Figure S5. SPLASH satellite tag location density maps for (a) MHI insular, (b) NWHI insular, and (c) open-ocean false killer whales. Grids are colored by the proportion of all locations for that population contained within each cell. Original (i.e., non-interpolated) filtered locations were used, and only locations that occurred within the time range of available behavior log data.


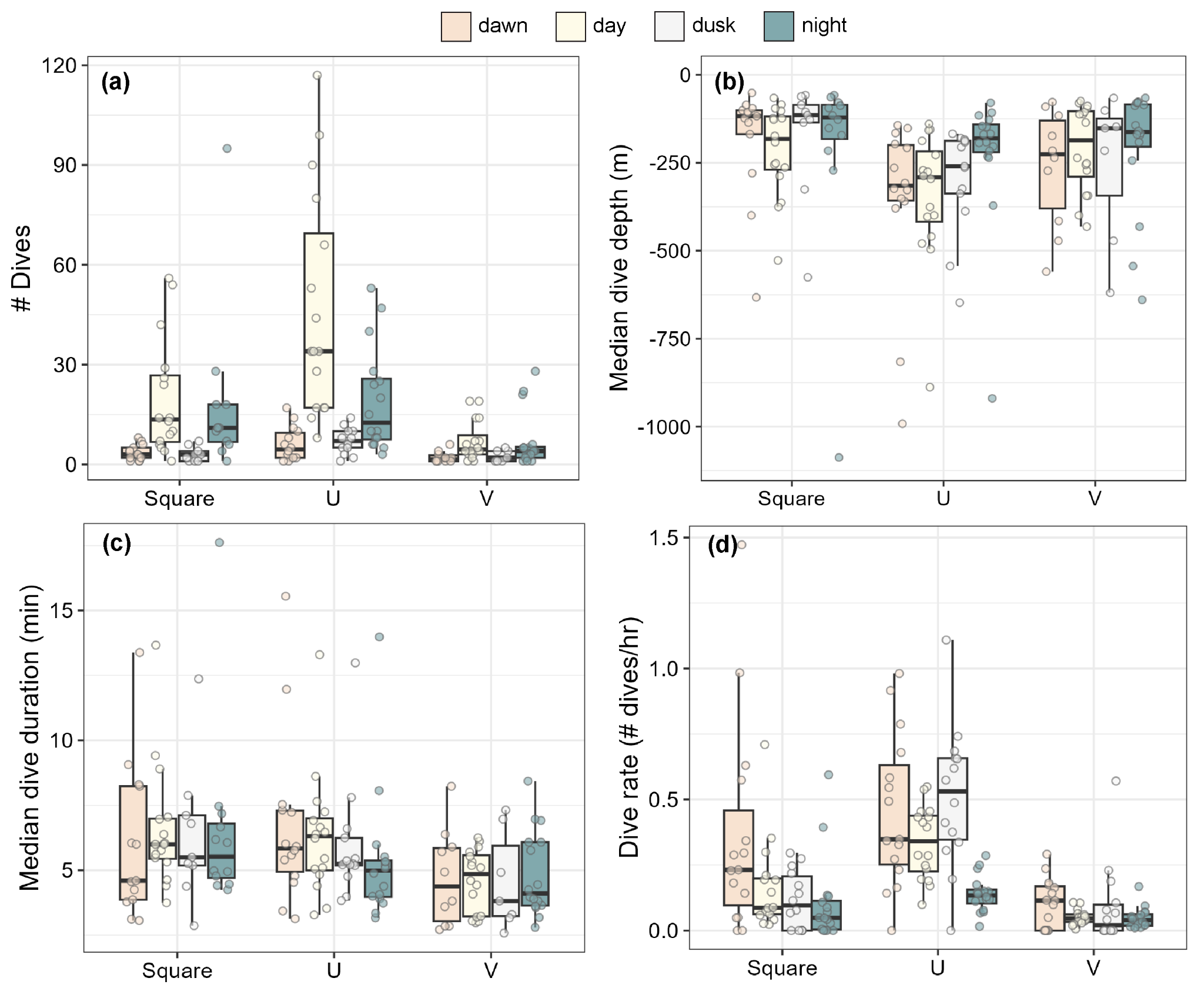


Figure S6. Dive metrics by dive shape and diel category; each jittered point represents a value for each individual (count or median of the individual). (a) the number of dives per category and shape; (b) dive depth; (c) dive duration; and (d) dive rate (number of dives per hour). All metrics represent dives 50 m or deeper and 2 min or longer. The middle line in each box represents the median, the lower and upper extents of the box the first and third quartiles (respectively), the lower and upper whiskers the minimum and maximum (quartiles +/- 1.5*interquartile range), and any values outside of the box/whiskers are shown as points.


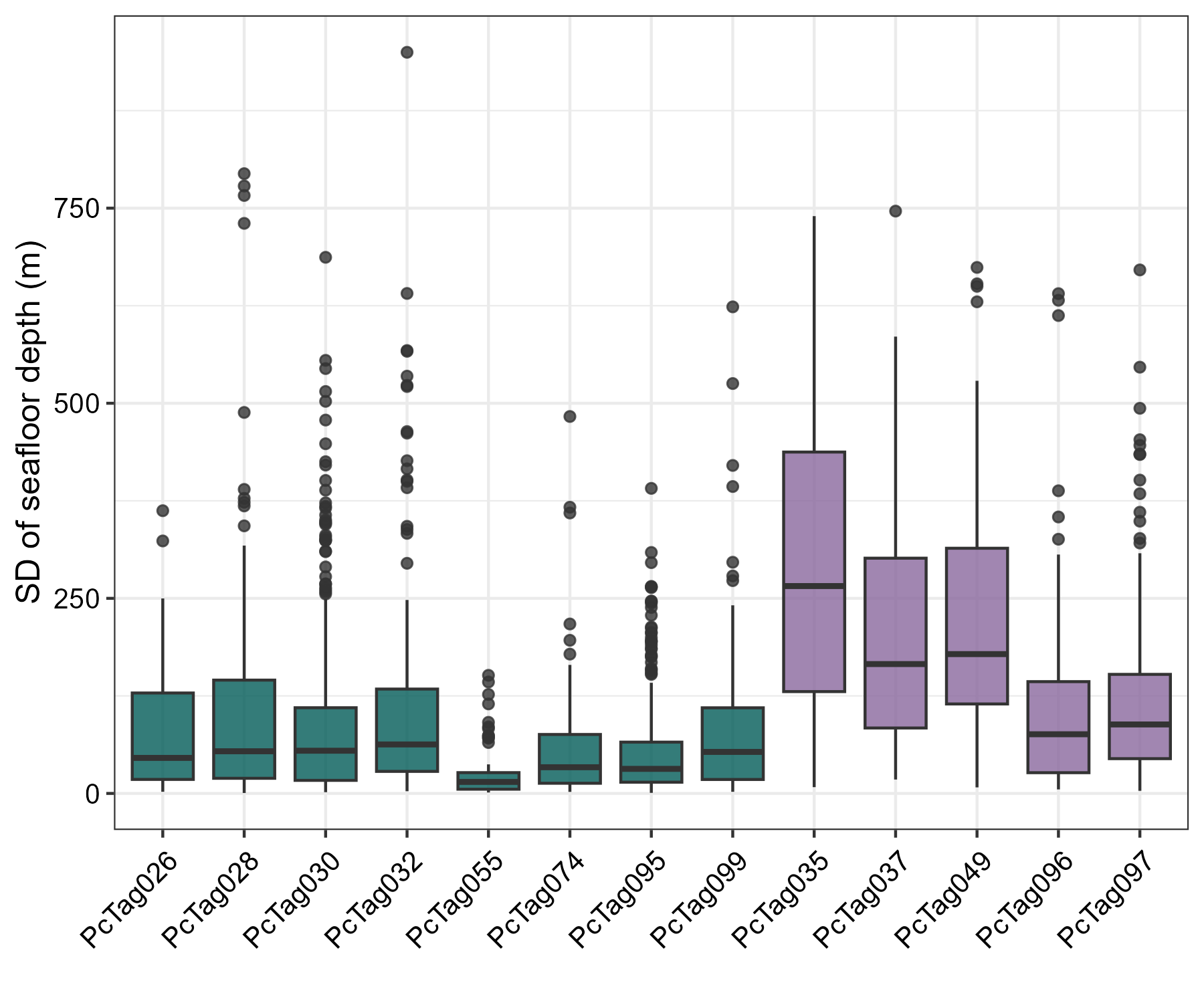


Figure S7. Distribution of standard deviation in seafloor depth values (calculated across all 20 imputed dive positions) for all dives with less than 4 km of positional uncertainty by insular false killer whales, indicating that many are likely an artifact of positional uncertainty rather than truly benthic diving behavior. Boxplots are colored by population assignment (green = MHI; purple = NWHI). The middle line in each box represents the median, the lower and upper extents of the box the first and third quartiles (respectively), the lower and upper whiskers the minimum and maximum (quartiles +/- 1.5*interquartile range), and any values outside of the box/whiskers are shown as points.


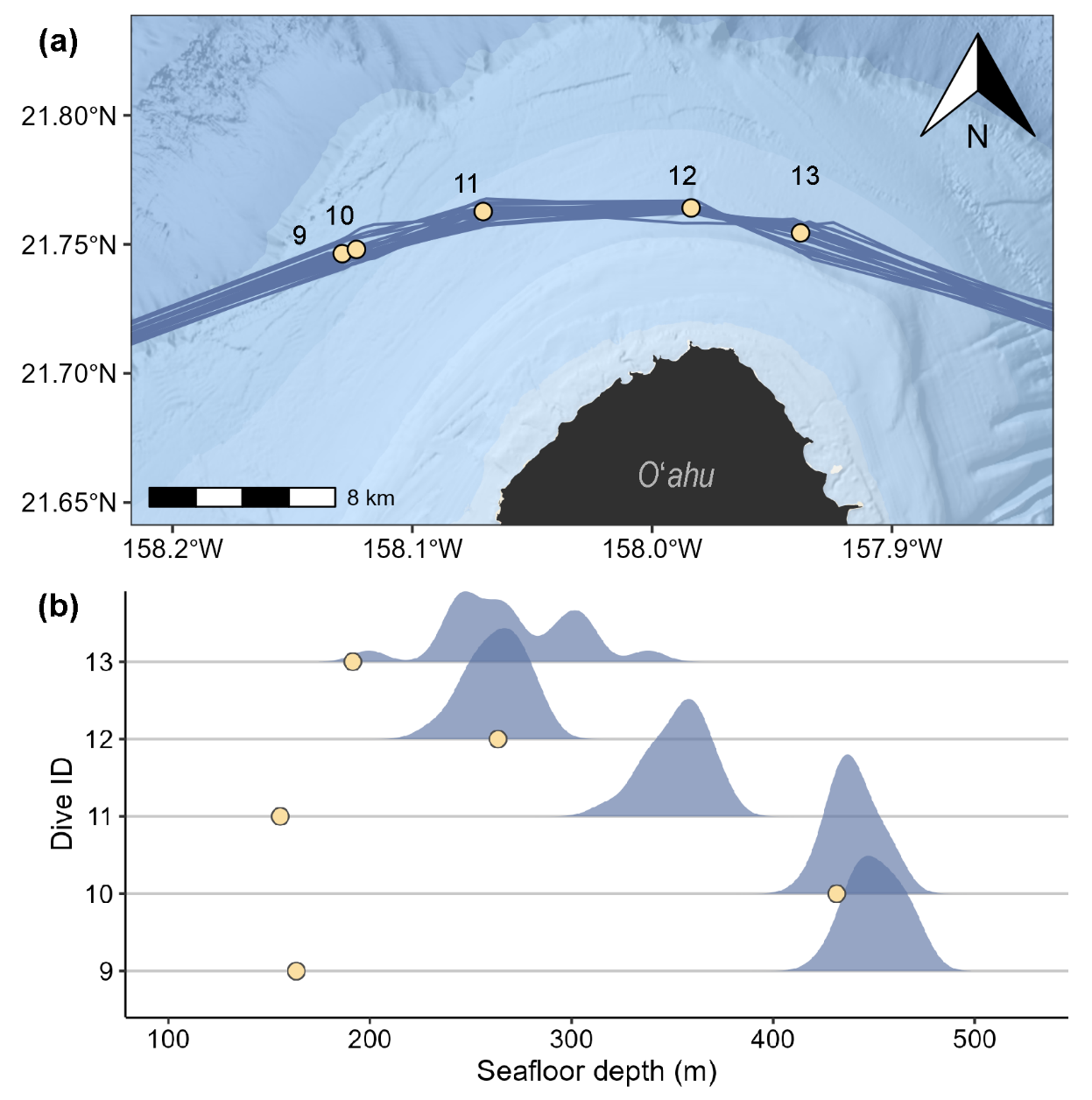


Figure S8. Example of using multiple imputations of dive locations to assess relationships between dive depth and seafloor depth using data from false killer whale tag deployment PcTag074. (a) Map of locations of five dives with estimated locations (yellow circles) and the 20 imputed tracks (gray lines). (b) Density plots of seafloor depth values across the 20 imputed dives (blue shaded density curves) and the recorded dive depth (yellow circle) for each of the dives shown in panel (a). Dives 9 and 11 were much shallower than the seafloor depth, while the remaining dive depths overlapped with their seafloor depths; dives 10 and 12 are probable seafloor dives, while dive 13 has higher uncertainty.


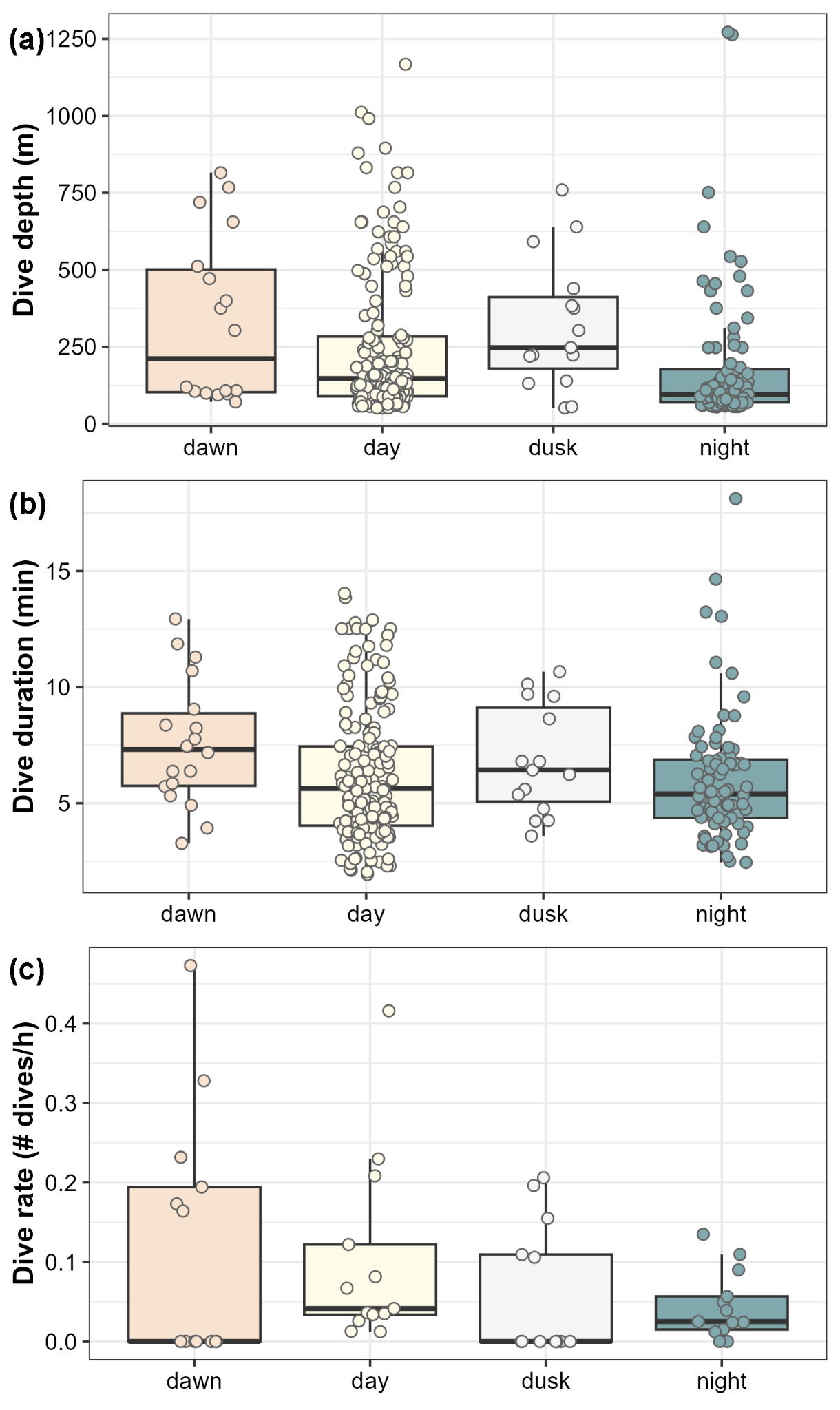


Figure S9. Distribution of (a) dive depths, (b) durations, and (c) rates of probable near seafloor dives by diel period for MHI and NWHI insular false killer whales. For dive rates (c), each point represents a single individual (as in Figure 5a), and thus the number of points across diel categories is the same in this panel. The middle line in each box represents the median, the lower and upper extents of the box the first and third quartiles (respectively), the lower and upper whiskers the minimum and maximum (quartiles +/- 1.5*interquartile range). Data points are plotted as jittered points.
